# Ethnopharmacological Field Study of Three Q'eqchi Communities in Guatemala

**DOI:** 10.3389/fphar.2018.01246

**Published:** 2018-11-06

**Authors:** Jorge Mario Vargas, Adolfo Andrade-Cetto

**Affiliations:** Laboratorio de Etnofarmacología, Facultad de Ciencias, Universidad Nacional Autónoma de México, Mexico, Mexico

**Keywords:** ethnopharmacology, ethnopharmacological field study, medicinal plants, traditional medicine, Q'eqchi

## Abstract

Mesoamerica is well known for the Mayan civilization, which flourished in this region during pre-Columbian times and made use of plant diversity for medicinal purposes. Currently, there are 21 Mayan ethnic groups in Guatemala, including the Q'eqchi'. The use of medicinal plants is still prevalent among them, they have been an important medicinal source for the population. The present study aims to compile traditional knowledge of the use of medicinal plants from three Q'eqchi' communities in Alta Verapaz, Guatemala and identify the important medicinal plants that are currently being used to treat relevant diseases. The study also aims to determine the relative importance of the identified species to propose new species for further pharmacological studies. Based on the cultural richness and the low level of perturbation of the vegetation, we selected the Q'eqchi' communities of Sanimtaqá, Santo Domingo de las Cuevas, and Chirrepec in Alta Verapaz, Guatemala. There, semi-structured interviews were conducted between June 2013 and December 2014 with common people. Plant-related questions and certain sociocultural contexts of the informants were included. Herbarium specimens for identification were collected with the help of the informants in their gardens with people from each community. The data were analyzed in two forms, the first non-quantitative based on the interpretation of the interviews (emic concepts of diseases) the second by following quantitative methods: informant consensus factor (Fic), Friedman's fidelity index (Fl), and use-reports (Ur). A total of 169 interviews were conducted. One hundred thirty-seven species of plants with medicinal uses were identified, which were described 2,055 times. These species belong to 59 families and 117 genera. Gastrointestinal conditions and pain/fever had the highest number of plant species uses for treatment. The main gastrointestinal conditions included diarrhea (Nume'sa'), stomach pain and worms (Luqum), while the pain/fever classification included headaches (rail jolom), and fevers (Tiq'). The most important cultural condition is called Chaquiq'yaj, the symptoms of the disease; diarrhea, vomiting, fever, lack of appetite, and thirst could be associated with a gastrointestinal. Conclusions: After analyzing the data, we can conclude that; *Ageratina ligustrina, Catopheria chiapensis, Baccharis inamoena, Peperomia maculosa, Baccharis salicina, Clinopodium brownei, Calea integrifolia*, and *Smallanthus maculatus* var. *maculatus* are the most culturally relevant species.

## Introduction

The use of medicinal plants is part of the traditions of every country and involves practices that have been handed down from generation to generation. The acceptance of traditional medicine by a population is largely dependent on cultural factors, and therefore, the majority of traditional medicine may not be easily transferred from one culture to another (Akerele et al., 1991).

Mesoamerica is well known for the Mayan civilization, which flourished in this region during pre-Columbian times. Guatemala, as part of Mesoamerica, is one of the places where some of the most important Mayan cities were established. The indigenous peoples who now live in this region have their origins in this civilization. Currently, there are 21 Mayan ethnic groups in Guatemala, of which the Q'eqchi' group is distinguished from other groups by its language of the same name, among other features (see section Brief History of the Communities below). The area of distribution of this group is divided between the lowlands of the Peten and the lands of the North Altiplano, including Alta Verapaz, Baja Verapaz, and Quiché (Quezada et al., 2008).

In Guatemala, the use of medicinal plants is prevalent because these plants and their associated traditional practices account for the main primary health care treatment modalities for the population, 54% of which lives in poverty (Instituto Nacional de Estadística, 2014). Based on WHO data for Latin America, we estimated that the mortality rate for children is 3–4 times higher than the national average in the indigenous population. In Guatemala, the following causes of death of children <5 years in age were reported: acute respiratory infections, 16%; asphyxia at birth, 14%; premature birth, 13%; congenital anomalies, 12%; lesions, 9%; neonatal sepsis, 9%; diarrhea, 8%; and HIV/AIDS, 1% (World Health Organization, 2012).

The use of medicinal plants by the Q'eqchi' of Guatemala has been the subject of previous studies using different approaches, some of the most recent being studies on a specific species to treat the culture-bound illness “susto” (Mullally et al., 2016), the antifungal activity of a single species (Ta et al., 2016), the diagnosis of the single culture-bound illness “emplotment” (Hatala and Waldram, 2017), the investigation and review of Q'eqchi women's reproductive health in the Lake Izabal region (Michel et al., 2016), the broad study of Mayan phytomedicine in Guatemala and a further proposal for patient-centered boundary mechanisms to foster intercultural partnerships in health care (Hitziger et al., 2016, 2017). The present study differs from these previous studies because it was conducted in three communities of Alta Verapaz with a normal population (no traditional healers).

The estimated population size of the department of Alta Verapaz in 2012 was 1,147,593 inhabitants, of which 90% were identified as belonging to the indigenous Q'eqchi' population. This department is considered rural because 77% of its inhabitants live outside an urban area. In 2011, the national poverty level reached 54%, while the poverty level in Alta Verapaz was 78%. In the same year, the primary-school enrollment rate was 100%, while enrollment at the basic level dropped to 43.5%, and that at the diversified level dropped to 20%; these data confirm that schooling in Alta Verapaz occurs mainly at the primary level (Instituto Nacional de Estadística, 2014).

In this region, the people face poverty and a lack of access to health-related resources. For these reasons, the use of medicinal plants remains an alternative and has high cultural value for the population. These facts, together with the presence of the native Q'eqchi' population, made this area ideal for an ethnopharmacological field study.

The present study aimed to compile traditional knowledge of the use of medicinal plants among non-specialists from three Q'eqchi' communities in Alta Verapaz, Guatemala, to identify the important medicinal plants that are currently being used to treat diseases, and to determine the relative importance (see below) of the identified species to contribute to the understanding of traditional medicine used by local participants.

To achieve this goal, information was analyzed in two ways. The first was a non-quantitative method based on the interpretation of interviews (emic concepts of diseases), and the second utilized the following quantitative methods: informant consensus factor (Fic), Friedman's fidelity level (Fl), and use reports (Ur) (Andrade-Cetto and Heinrich, 2011).

This methodology allowed us to obtain reliable information about the categories of diseases and the main diseases that are treated with medicinal plants, the fidelity of the informants regarding the use of a species for the treatment of a disease and the number of use reports for each species as an indicator of the frequency of mentions. In terms of research questions, the approaches described in the above paragraph allow us to postulate the following questions: (A) Is there a consensus between the informants if we analyze the diseases by category? (B) What are the main diseases, and how are they treated? (C) Do the informants appreciate the use of a species for the treatment of one illness or symptom? (D) Is there a culturally-based syndrome or condition that can be biomedically correlated? (E) Has a correlation with plant use been reported in previous studies in the area?

The results of the analysis provided information about species that are most likely to have biological activity in subsequent pharmacological studies. In addition, socioeconomic data were included to establish the social context of the studied communities.

The present study was designed in 2013, and the recommended standards for conducting and reporting ethnopharmacological field studies (Weckerle et al., 2018) were adapted as much as possible.

## Methods

### Community selection

Based on cultural richness, the low level of vegetation perturbation and the lack of previous specific studies in the towns, we selected the Q'eqchi' communities of Sanimtaqá, Santo Domingo de las Cuevas and Chirrepec in the department of Alta Verapaz, Guatemala (see Figure S1). The type of vegetation in this region is a cloud forest (Ministerior de Agricultura Ganadería y Alimentación, 2002), with an average elevation of 1,323 mamsl, an average temperature between 17.4 and 20.1°C, rainfall between 1,589 and 2,842 mm and a relative humidity of 78.89% (INSIVUHME, 2009). The present study followed the strategy (interviews and analysis of results) used by Andrade-Cetto (2009) in the community of Tlanchinol, Hidalgo, Mexico.

The abovementioned communities were chosen based on the definition of indigenous people given by Montenegro and Stephens (2006), who defined indigenous people as the descendants of the original colonizing inhabitants and those who have been accepted by and live in the community; the members of the chosen communities are all Q'eqchi' and have colonized the area for several generations.

To comply with the regulations of the local government for the protection of biodiversity and institutional ethics protocols, Free Prior Informed Consent (PIC) was acquired directly from the Federation of Cooperatives of the Verapaces (FEDECOVERA), who are the legal representatives of these communities.

In the present study, we focus on people who use medicinal plants daily but are not considered traditional healers. One reason underlying the use of medicinal plants is the health service situation in the studied communities. In Sanimtaq'a, there is no pharmacy, and in Santo Domingo, there is no health center or pharmacy. In Chirrepec, a small house is used as a health center. In Chirrepec and rarely in Sanimtaq'a, health ministry personnel come to see children under 5 years old and pregnant women, mainly to administer vitamins, treatments for diarrhea and respiratory diseases and vaccines for diseases such as measles, chicken pox, and smallpox.

### Brief history of the communities

At the end of the Mayan Classic period, which was influenced by Chich'en Itzá, a new network of commercial exchange formed between lineages from the coast of the Gulf of Mexico. In the Mayan Post-classic period, this commercial network of Mayan groups from northern Yucatan and the Transversal Strip of northern Guatemala spread to southern Petén through the Usumacinta, La Pasión, and Salinas rivers, as well as the Mopán watersheds. In the colonial times of the sixteenth century, the Dominican friars in Verapaz settled in smaller groups of the descendants of these lineages, now known as the Q'eqchi'es. These groups include Cobán, Carchá, Chamelco and San Cristobal Verapaz (Van Akkeren, 2012).

Many of the expressive forms of the Q'eqchi' culture, such as the costumes, dances, music, brotherhoods and veneration to the saints, are products of the colonial era. However, the basis of the Q'eqchi' identity that stems from concepts of the ethnic community and its borders proposed by Max Weber and Fredrik Barth (Camus, 2006) is built upon its worldview (its relationship with nature and religious beliefs), which is considered to be rooted in the common past of the Q'eqchi'. Within this worldview, the Q'eqchi' are communities anchored to their relationship with the land (the community where they live). Belonging to a locality is important in Q'eqchi' communities, since many identify with a particular village or locality (Richard, 1993). This relationship with the land highlights the cult of Tzuultaq'a (Valley-Hill god) and the celebration of mayejak, which, among other things, is the rite of offerings and sacrifices to Tzuultaq'a for a good harvest of corn (Richard, 1993; Cabarrús, 1998; Wilson, 1999; Estrada Ochoa, 2006; Cahuec del Valle Eleuterio, 2009; Schackt, 2012; Caballero Mariscal, 2013).

In Guatemala, Q'eqchi' is the fourth most commonly spoken language, with the greatest number of speakers (more than 700,000) after Quiché, Mam and Kaqchiquel. Q'eqchi' are separated from other Mayan speakers and are mainly found in Alta Verapaz, Izabal, Quiché and southern Belize. This was not the case at the time of the Spanish conquest, when the highest distribution of the Q'eqchi' population was in Coban, San Juan Chamelco, Carchá, Cahabón and Lanquín, which are all in Alta Verapaz. The migratory trend from the highlands to the lowlands began in 1870 when President Justo Rufino Barrios encouraged the migration of Europeans to Guatemala to exploit the potential of crops such as coffee (Zarger, 2002).

Coffee was introduced in Guatemala in the 1740s, and coffee production boomed in 1860, with coffee becoming the main export product, replacing cochineal (insect; Dactylopius coccus, from which the natural dye carmine is derived), which had been the predominant product marketed in the years after 1821 (Wagner, 2001). The communities that are part of this study currently reside in what were previously coffee farms owned by Germans who came to Alta Verapaz attracted by the low prices of land and the large amount of Q'eqchi labor inhabiting these lands. By 1883, the Sanimtaqá and Santo Domingo de las Cuevas communities belonged to the Samac estate, which was owned by August and Gustav Helmrich. The community of Chirrepec was owned by Georg Boehm and later became a tea estate under its new owner, Oscar Majus Koefler. Following the declaration of war by Guatemala on Germany at the end of 1941, under pressure from the United States, Guatemala proceeded to gradually intervene, expropriate and nationalize German property, while Germans were deported to the United States or repatriated to Germany. In 1952, the law of agrarian reform was promulgated in Guatemala, where several farms expropriated from Germans were given to cooperatives. In 1976, the FEDECOVERA was formed, grouping all the coffee farms expropriated from the Germans in Alta Verapaz (Wagner, 2001). In this sense, the heritage of the communities being studied and that of other communities was returned to the members of these communities, who had occupied these territories prior to the arrival of the Germans.

### Communities

#### Community of sanimtaqá

The community of Sanimtaqá, which belonged to the Samac estate, is a Q'eqchi' community consisting of 54 families in a cooperative. This community is located 10 km west of Cobán, the departmental head of Alta Verapaz (Figure S1), at a latitude of 15°19′16.6^′′^ north and a longitude of 90°27′48.4^′′^ west. The name Sanimtaqá, meaning “deep,” comes from Q'eqchi' and refers to the location of the community, which is at the bottom of a basin. The terrain is very steep, and the relief possesses karstic characteristics. The type of vegetation is a cloud forest. It is believed that the settlers of this community came from the city of Carchá, which is ~18 km away. The main source of income for this community is the cultivation of coffee and cardamom. In addition, the women of the community weave handicrafts to sell. The cultivation of beans and corn is for sustenance.

#### Community of santo domingo las cuevas

The community of Santo Domingo las Cuevas comprises ~30 families, 13 of which live in a cooperative. All the families come from the community of Samac, a former coffee farm located 2 km away (Figure S1). This community is located 8 km from Cobán, at a latitude of 15°27′39.98^′′^ north and a longitude of 90°25′51.03^′′^ west. The terrain has a pronounced slope between 20 and 50%. The relief is steep and has karstic characteristics, and the type of vegetation is a cloud forest. The main sources of income for this community are pine cultivation and the sale of labor in Cobán. The farming of beans and corn is mainly for subsistence agriculture.

#### Community of chirrepec

The community of Chirrepec consists of 250 families in a cooperative. This community is also located 8 km from Cobán, in the municipality of San Juan Chamelco (Figure S1), at a latitude of 15°25′52.2^′′^ north and a longitude of 90°20′59.1^′′^ west. The slope of the land is between 10 and 20%. The relief is steep with karstic characteristics, and the type of vegetation is a cloud forest. The main sources of income for this community are the cultivation of tea (*Camelia sinensis* L.), the sale of labor in the cooperative that operates in the community and the sale of labor in Cobán. The farming of beans and corn is for sustenance.

### Interviews

Due to our difficulties in speaking and understanding the Q'eqchi' language, a community member helped us to translate and increase trust. Initial visits to each house were extensive and were always made by the first author using free-listing (Weller and Romney, 1988) and recording; medicinal species, local names, documented uses, preparations, recipes, and other information was obtained at the first visit, during which the previously obtained PIC was explained to each participant. After that, several days were spent in semistructured interviews (Bernard, 2006) at each house.

From the interviews, information was obtained about knowledge of the main medicinal plants used by the informants. Questions about certain sociocultural contexts of the informants were also included. Some of the questions regarding knowledge of medicinal plants were as follows: the common name (Q'eqchi') of the plant, perception of the form of the plant, part of the plant that is used, disease for which the plant is used, and preparation and application of the medicine (see Table S1). The interviews were conducted in all three communities. The first author visited the communities 5–7 days monthly (from June 2013 to December 2014). All interviews were conducted in Q'eqchi', and each interview lasted between 0.5 and 1.5 h, depending on the knowledge and the degree of collaboration of the informants. A total of 169 semistructured interviews were conducted, of which 21% were conducted in Sanimtaqá, 7% in Santo Domingo las Cuevas, and the remaining 71% in Chirrepec.

### Plant collection

With the permission of the FEDECOVERA and of each individual cooperative, herbarium specimens for identification were collected with the help of the informants in their gardens in Sanimtaqá and Chirrepec and in the forest with informants from each community. The specimens were deposited at the BIGU herbarium of the School of Biology of the Faculty of Chemical Sciences and Pharmacy, as well as the AGUAT herbarium of the Faculty of Agronomy, both of the University of San Carlos de Guatemala.”

### Data analysis

As the result of direct observations (participant observation), we present descriptive data in tables and in the results section; the information was analyzed in a non-quantitative manner based on the interpretation of the interviews (emic concepts of diseases), with the aim of identifying the plants heavily used for a certain ailment or disease category. Quantitative tools were used to analyze the results regarding the general use of plants; specifically, we used the informant consensus factor (Heinrich et al., 1998). This factor was originally used to highlight plants of intercultural relevance and agreement in the use of plants. To use this tool, it was necessary to classify the illnesses into broad disease categories (several diseases based on the organ systems in one category), as follows: (1) Gastrointestinal, (2) Respiratory, (3) Pain/Fever, (4) Dermatological, (5) Muscular/Skeletal, (6) Cardiovascular, (7) Urological, (8) Diabetes, (9) Reproductive, (10) Cultural Filiations, (11) Oncologic, and (12) Others. As result of this analysis, it was possible to evaluate whether there was agreement in the use of plants in the illness categories between the informants.

The Fic was calculated as the number of use citations in each category (nur) minus the number of species used (nt), divided by the number of use citations in each category minus one:

$${\text{F}}_{\text{IC}}\;=\frac{n\text{ur}-n\text{t}}{n\text{ur}-1}$$

The fidelity level (Fl), which is the ratio between the number of informants who independently suggested the use of a species for the same major purpose and the total number of informants who mentioned the plant for any use, was calculated for the most frequently reported diseases or ailments for the categories with the highest Fic values:

$$\text{Fl}\;(\%)\;=\frac{Np}{N\times 100}$$

where Np is the number of informants that claimed the use of a plant species to treat a specific disease, and N is the number of informants that used the plants as a medicine to treat any given disease. The aim of the FL is the arrangement of species in accordance with the percentage of informants suggesting the same medicinal use for a given species compared with the total number of informants reporting any sort of use for that plant (Friedman et al., 1986).

We defined use report (Ur) as the number of informants that report the use of a species for a specific disease (Heinrich et al., 1998; Andrade-Cetto, 2009).

With the help of these tools, we determined which illness categories had more “consensus” regarding plant use (Fic) and the plants with major fidelity among these categories (Fl).

## Results

### General analysis of the data

After plant recollection in the communities, taxonomic work was performed, allowing us to determine 137 species with medicinal uses, which were associated with 2,055 mentions of use; the species belonged to 59 families; and 117 genera. All scientific names are given according to The Plant List: http://www.theplantlist.org (Table S1).

### Social aspects community of sanimtaqá

Thirty-six informants in this community were interviewed (one individual per family). The houses in this community are made of wood and have laminated roofs. The houses have latrines and drinking water but do not have electricity. The orchards and gardens contain ornamental and medicinal plants. The community must be accessed on foot since the road is in poor condition.

Of the people interviewed, 36% were women, and 64% were men. Sixty-six percent of the interviewees did not speak Spanish, and 33% spoke Spanish but with difficulty. The ages of the informants ranged from 20 to 80 years. Most of the interviews were conducted with people between 30 and 50 years of age (55%). Only 27% of the interviews were conducted with seniors over the age of 50. Of the total number of interviewees, only 30% could read and write, with education levels ranging from the third grade of elementary school to the sixth grade.

When asked how they learned the tradition of using medical plants, 83% of the informants mentioned that they learned this tradition from their parents and grandparents, and the rest (17%) of the informants were either self-taught or learned this tradition from friends. Some of the informants commented that young people prefer to buy medicines at the pharmacy to avoid having to go out to collect the plants. The informants also mentioned that the use of medicinal plants becomes more relevant when they do not have money to buy prescribed pharmaceuticals. Most of the interviewees over 50 years of age mentioned that medicinal plants were the only healing alternative that their parents had when they were children, since pharmacies and health centers were either sparse or absent.

During the interviews, four ways in which informants cure their illnesses were detected: first, they use their own knowledge of medicinal plants for healing; second, they look for a pharmacy and ask what they can buy to cure their illness; third, they look for a healer who prescribes medicinal plants; and fourth, when none of the above options work, they visit hospitals or health centers. The traditional way by which the interviewees obtain medicinal plants is by collecting them from gardens, especially exotic plants that they have obtained from friends, or from the market. The informants also collect plants that the community considers medicinal from the roads of the community and from the edges of fields. Finally, the forest is a source of medicinal plants; the informants look in the forest for plants that are not found in sites that have been disturbed. Forty-six percent of plants are gathered along small roads.

#### Community of santo domingo las cuevas

Approximately 30 families live in this community, with an average of 4.5 people per family. Of the 30 families, 13 belong to a cooperative; these were the families interviewed. Most of the houses in this community are made of wood, have laminated roofs and have not been floored. The houses have drinking water, latrines, and electricity. Access to the community is by a dirt road that is in good condition. Being 20 min from an urban center, this road is regularly used by public transport and vehicles.

Twelve informants were interviewed (one informant per family). The cooperative is 17 years old; all families had belonged to the Samac estate, and relatives of the informants had lived in Samac since before their grandparents, who were already living at that site before the arrival of the Germans in 1870. This community is dedicated to the cultivation of coffee, the sale of wood and the sale of labor. Of the people interviewed, 42% were women, and 58% were men. Fifty-eight percent of the interviewees did not speak Spanish, and 42% spoke Spanish but with difficulty. The ages of the informants ranged from 30 to 80 years. Of the total number of interviewees, only 25% could read and write, with education levels ranging from the third grade of elementary school to the third elementary level.

When asked how they learned the tradition of using medical plants, 91% of the informants mentioned that they learned this tradition from their parents and grandparents, and the rest (9%) were either self-taught or learned this tradition from friends. Some of the informants claimed that they use medicinal plants because it is cheaper to do so and felt that they should go collect the plants themselves. Several families search for pharmacies because it is easier to cure illnesses with pills; however, they also consult healers when they do not know how to cure their ailments or, eventually, look for a hospital or a physician. Medicinal plants are obtained from gardens and neighboring roads and from the mountains.

#### Community of chirrepec

This community is composed of 188 families, with an average of 6 people per family. A total of 120 informants were interviewed (one per family). The houses of 62% of the informants are made of wood, have laminated roofs and have not been floored. The houses of the remaining 38% are made of concrete and have laminated roofs and floors. Drinking water and electricity are available throughout the community. The orchards or gardens of the community contain ornamental and medicinal plants. Access to the community is by paved road, so any type of vehicle can access the community. Most families have lived in the community since before their grandparents, who were already living at that site before the arrival of the Germans. This community has been dedicated to the cultivation of tea since its previous ownership by Oscar Majus Kofler, a German whose land was expropriated in World War II.

Of the people interviewed, 24% were women, and 76% were men. Forty-six percent of the interviewees did not speak Spanish, and 54% spoke Spanish but with difficulty. The ages of the informants ranged from 18 to 82 years. Most of the interviews were conducted with people 50 years or older (43%). Twenty-seven percent of the interviews were conducted with people aged 18–30 years, and the remaining 29% with people aged 30–50 years. Of the total number of interviewees, 43% knew how to read and write, with education levels ranging from the third grade of elementary school to bachelor's and accounting degrees.

When asked how they learned the tradition of using medical plants, 92% of the informants mentioned that they learned this tradition from their parents and grandparents, and the rest (8%) of the informants were either self-taught or learned this tradition from friends. Some of the informants commented that most young people prefer to buy medicines at the pharmacy to avoid having to go out and collect the plants. People over 50 years of age learned this tradition from their parents and grandparents because it used to be the only way to treat illnesses, as there were no pharmacies or physicians to consult, only healers. The traditional way to learn how to identify and use medicinal plants is for the child to go with the father to the agricultural land, where the child is shown medicinal plants that should not be cut. At home, girls learn from their mothers how to prepare and use medicines from the plants. The best time to learn the use of medicinal plants is upon marriage, as both men and women need to heal their children when they cannot afford to go to a physician or pharmacy.

The main sociocultural aspects of the communities are presented in Table 1.

**Table 1:** Summary of the socio-cultural aspects of the communities, + indicates prescense.

**Concept**	**Sanimtaq'a 36 interviews**	**Santo Domingo 12 interviews**	**Chirrepec 108 interviews**
Number of families	54	13	188
Inhabitants per family	4	4.5	6
Number of informants	36	12	120
Access to the community		
Dirt roads in poor condition	+	
A dirt road in good condition		+
Pavement			+
Type of houses		
Wood, laminated roofs and floor	+	+	+
Block, laminated roofs and floor	+	+	+
Access to public services		
Water	+	+	+
Light		+	+
Telephone		
Cable TV		+	+
Subsistence crops		
Beans and maize	+	+	+
Major crops		
Coffee	+	+
Cardamom	+	
Tea			+
Wood		+
Other sources of income		
Sale of labor		+	+
Total of men interviewed	64%	58%	76%
Total of women interviewed	36%	42%	24%
Speak Spanish	33%	42%	54%
Age of the informants		
0–29	23%		27%
30–50	55%	58%	30%
50 onwards	27%	42%	43%
Know how to read and write	30%	25%	43%
Schooling		
With no schooling	+	+	+
Primary	+	+	+
Basic		+	+
Diversified			+
Where they learned the use of plants		
Parents and grandparents	83%	91%	92%
Alone or with friends	17%	9%	8%
Health Options		
Self-medication with medicinal plants	+	+	+
Use of healers	+	+	+
Use of hospitals or doctors	+	+	+
Site where medicinal plants		
Garden	+	+	+
Communal Roads	+	+	+
Mountain	+	+	+
Time to live in the community		
0–25 years		+
100 or more years	+		+

### Preparation of medicinal plants

There are two main methods for the preparation of medicines from plants. First is the preparation of a decoction of tender leaves, which is then orally ingested; this method is mainly used for respiratory and gastrointestinal conditions. Infusion preparation is also used but is less common. Another form of preparation consists of heating the leaves of certain succulent and aromatic plants and then applying them as a poultice; this is mainly use for the topical treatment of muscular/skeletal conditions.

For the treatment of culture-specific conditions, baths are very common. There are two ways to prepare plants for baths. One uses plants considered to be of a “hot” nature, which are heated in water; these baths are performed with hot water. The other method uses plants that are “cold,” which are left to soak outdoors; these baths are performed in the early hours of the morning. For the treatment of dermatological conditions, sometimes the dry leaves are ground, and the powder is applied to the wounds; at other times, the juice of the epidermis of the stem is used to heal burns or wounds.

To better understand the preparation of each plant, we measured the amount of each plant (in g) used for each remedy in the field with the help of a portable scale (Table S1).

### Q'eqchi' concepts of the most common diseases

In Table 2, we summarize some of the emic concepts of diseases. The aim of this analysis was to correlate the concept of a disease of the interviewed people with a biomedical point of view. Diseases are culturally dependent, and the only difference is the labels used to describe disease. In some cases, there is a direct correlation with observed symptoms in which biomedicine and traditional medicine “agree,” but in other cases, there is an indirect link or no notable link.

**Table 2:** Q'eqchi' concept of the most common diseases found in the communities of Sanimtaq'a, Santo Domingo, and Chirrepec.

**Disease**	**Q'eqchi**
Abortifacient NMI	To assist in the delivery, or when woman want to abort a child, regularly is not given to pregnant women because they lose the child
Acne NMI	(Wa) are pimples or rashes occasioned by mudblood, mainly in young people. It is a disease
Amoebas MI	A kind of worm, stronger than common worms. Produces diarrhea and fever, feces are greenish yellow to white. Can produce vomiting
Anemia MI	(Maxil tib'eb') People who do not have enought vitamins, are weak, have fatigue, sleep and lack of strength. Can have hair loss
Antivenom NMI	(Xtiwom kanti') remedy for snake bite
Asthma MI	(Jiq') Dry cough without phlegm. The person has trouble breathing
Cramp NMI	(Much quej) pain in the muscle. When the body cools down it hurts the muscle. Also exits through a cold wind in the body
Itching NMI	(Wotz'oq) Itching in the skin
Depression NMI	(Xwajenaq) Sadness or colic. When a person gets angry and was very sad. May be caused by a bad experience a concern or nerves. Also called pain of heart (Ra'salinch'ol)
Diabetes MI	It is when the blood is sweet. It happens to people who are overweight, they after are thin. The person goes to the bathroom a lot and drink plenty of water. The body becomes something yellow. You can fall. Is produced by a scare or by eating too much candy
Dysentery NMI	(Ki'sa') Diarrhea with blood and pain. There are two types of dysentery; with blood and a lot of pain and white with less blood, Can cause fever
Body pain NMI	(Much quej) Produced by excessive physical work, charge a lot of weight, shock, or a flu
Muscle pain NMI	(Much quej) Pain in any part of the body, by excessive load, much physical work, or beating
Constipation NMI	(Xtzap Xsa') when a person is unable to have a bowel movement from lack of water or by disease
Fever NMI	(Tiq') sweating, feels discomfort and very cold
Grains NMI	(Xox) They can be big. caused by a dirty blood or infection in the skin, the skin can suppurate at some point
Bleeding in general NMI	When a lot of blood come out from a wound or menstruation
Wound NMI	(I'Q'ol) is given by beating, cuts. There are a lot of bleeding
Vaginal infection NMI	(Rahil Kub'sa') infection in the private parts of woman
Clean the blood NMI	(Xmesb'al Xsa'kik) Normalize the blood. Remove the dirt from the blood
Spots on the skin NMI	(Sa'lep) Occurs in children of 5–15 years due to lack of vitamins and weakness in the body
Mu NMI	A person who is not feeling well was attacked by a “bad wind,” the person feels uneasy, has a weakness, headache, vomiting. Also, it is said when the soul of a dead person does not come out of the house because it was a bad person the soul remains in the home and among his acquaintances
Red Eyes NMI	(Raa hu') Conjunctivitis
Purgative NMI	Medicine to clean the stomach through bowel movements
Burns NMI	(Q'atal) When the skin is affected by contact with something warm
Colds NMI	(Wosol), flu-like illness. Symptoms such as a sore throat, cough, runny nose, and fever
Sweating In Children NMI	(Saq'tiq'ob') Sweating
Sprain NMI	(B'achal) resentful body by a bad move. There is no swelling
Varises NMI	(Ich'mul na sipo) Inflammation of the veins, mainly manifests in the legs
Stomach acidity NMI	Heartburn, it may be by gastritis
Alopecia NMI	Fall of hair for using harsh soaps, an ill, concerns, malnutrition or inherited
Blisters NMI	(Pox has') bumps that come out after a burn
Anxiety NMI	(Maxil wank) Restlessness, people not calm
Arthritis MI	(Moch quej) pain and swelling in the joints. The affected part is inmobil
Awas NMI	It is a disease that comes out in newborn babies because the mother stayed with the desire to eat something and the child comes up with the skin with something similar to that
Chakiq yaj NMI	Also called hijio. Regularly happens to children, the symptoms are: weeping, whitish eyes, fever, diarrhea, chills, loss of appetite. It is a disease. It is cured with a cold water bath with herbs. When the child is bathed with cold water very early in the morning, water vapor comes out of the body, it is known that the bath have a good effect. Regularly the bath is done three times in 3 days. The child must take a bervage of herbs before the bath
Weakness NMI	Lack of vitamins, not eat well, or for a lot of work, or by disease
Dermatitis NMI	(Wotz'oq) Itching in the skin
Diarrhea NMI	('Sa') Appears when the food is not digested well. It is also manifested by going to the bathroom frequently, and defecate watery. You may have vomiting
Headache NMI	Headache
Toothache NMI	Pain of teeth with caries
Post-partum Pain NMI	Pain after delivery. It can occur throughout the body or in the womb
To facilitate the delivery NMI	Help a woman to give birth with plants; “mesbe” or “ruk max,” helps to have a rapid delivery. Sometimes happens when they run out of amnionict fluid
Gastristis MI	Burning in the stomach for eating or drinking irritating things. It can also occur when you have a disorder in the mealtime
Flu MI	(Ojb') stuffy nose, runny nose, sore throat, fever, cough, sore ears, red eyes, body aches and headache. It is similar to the cold
Hepatitis MNI	When a person turns yellow or pale and the has a lot of weakness
Infection in the throat NMI	(Rahil Xsa'kux) When you feel pain in the throat
Inflammation NMI	(If pook) Swollen by any wound. Regularly produces pain and acquires reddish color
Worms NMI	(Luqum) Disease that gives by dirt. Ssymptoms belly swollen, diarrhea, vomiting, loss of appetite and stomach pain
Menstruation NMI	(Puch'unik) moment that comes to the woman, vaginal bleeding at this time
Clogged Ear NMI	(Tzap xik) Ear that it was cover by wax or by a garbage
Mumps MI	Inflammation of the lymph nodes in the neck or thigh with fever, headache, and lack of appetite
Broken Bone	(Toqol) Broken Bone
Reflux	Burning of chest and throat after eating a lot, mainly in the evening hours
Measles MI	Disease that causes pimples all over the body of the child, causes fever
Fright NMI	(Xuwajenaq) When the person saw something or something bad happens. The person feels uneasy, doesn't want to work, does not sleep, he has a weakness, has nightmares
Cough NMI	(Qux) dry cough and phlegm. Causes difficulty breathing and discomfort in the throat

*MI we assume needs medical intervention for diagnosis. NMI, we assume people can detect it without medical intervention*.

We can divide these concepts into three categories:

1) The terminology is adapted from the “western” medicinal point of view, such as gastritis, mumps or diabetes. Normally, such a diagnosis requires a biomedical examination and diagnosis, while in a traditional system, certain signs may point to a disease condition, but it is not possible to directly correlate the traditional with the biomedical concept. For example, the word diabetes is used with the definition “it is when the blood is sweet,” and possible causes and observed symptoms are described, but these concepts have nothing to do with accepted definitions, such as the ones given by the World Health Organization, the American Diabetes Association or the International Diabetes Federation. For these agencies, insulin resistance is a key factor to understand the disease. Therefore, biomedical terminology is used, but conceptually, there is no clear link between the cultural understanding of the disease and the disease according to biomedical concepts.
2) Diseases or “symptoms” can be diagnosed by people based on the direct observation of the condition and correlated with “western” concepts such as worms, toothache, or vaginal infections. A medical intervention is not needed for diagnosis.
3) Diseases cannot be clearly and directly correlated with a biomedical disease concept. Further investigation is needed to clarify if such a correlation exists; for example, the terms “Chakiq yaj” or “hijio” are used when an individual presents the following symptoms: weeping, fever, diarrhea, chills, and loss of appetite, which regularly occur in children. Thus, could this observation be correlated with rotavirus infection? *In vivo* tests are needed to assess this possibility. In any case, “Awas,” “Chakiq yaj,” and “Fright” are not regularly detected by medical intervention (Table 2).

### Quantitative analysis

1. Informant consensus factor (Fic)
  Categories were formed based on the part of the human body affected by the disease, as per the methodology proposed by Andrade-Cetto and Heinrich (2011). The categories with the highest consensus in terms of the number of species used for treatment were gastrointestinal conditions and pain/fever, with an Fic of 0.89. The category of gastrointestinal conditions had 432 mentions and 47 associated taxa, while pain/fever had 376 mentions and 44 associated taxa, in agreement with our investigation question. We obtained results for the main categories of disease in which the informants had better agreement (see **Table 4**).
2. Plants with high use-report values (Ur) and high-fidelity levels (Fl), by category
  For this analysis, the categories with the greatest Fic values were included, and within each category, species with the highest fidelity levels (Fl) were included to highlight the most important species. Additionally, in terms of the choice of species, species with the highest number of use reports (Ur) were considered. To prevent species with only two or one mentions from being chosen over the most frequently mentioned species, these uncommon species were not included in the analyses. In the present work, we found that an analysis using Fl and Ur only works when the number of individual mentions for a species is large; for example, *Ageratina ligustrina* has more than 100 individual use reports for at least 6 different conditions, and most of them occur in the same category. The most prominent species by category are presented in Tables 3A,B.
3. Plants with high overall use-report values
  The plants with the highest overall use reports are presented in Table 3C.

**Table 3:** Analysis of the main plants used.

**(A) Plants with great use-reports**
*Catoferia chiapensis* (Ur; 155)
*Peperomia maculosa* (Ur; 111)
*Cymbopogon citratus* (Ur; 111)
*Ageratina Ligofilia* (Ur; 103)
*Citrus sinensis* (Ur; 92)
*Dysphania Ambrosoides* (Ur;89)
*Smallanthus maculatus* var. *maculatus* (Ur;73)
*Baccharis trinervis* (Ur; 60)
*Ocimum basilicum* (Ur; 54)
*Ruta graveolens* (Ur; 52)
**(B) Plants with high level of fidelity (Fl) and Use-reports (Ur) by category**
**Gastrointestinal**
*Ageratina ligustrina* (Fl 98, Ur 101)
*Dysphania ambrosoides* (Fl 88, Ur 79)
*Psidium guajava* (Fl 63, Ur 32)
**Respiratory**
*Baccharis trinervis* (Fl 34, Ur 20)
*Catoferia chiapensis* (Fl 10, Ur 16)
*Cymbopogon citratus* (Fl 22, Ur 24)
**Pain and Fever**
*Baccharis trinervis* (Fl 32, Ur 19)
*Catoferia chiapensis* (Fl 78, Ur 122)
*Coffea arabica* (Fl 43, Ur 20)
*Cymbopogon citratus* (Fl 77, Ur 86)
**Dermatological**
*Calea integrifolia* (Fl 100, Ur 21)
*Salmea scandens* (Fl 43, Mu 21)
*Smallanthus maculatus* var. *maculatus* (Fl 93, Ur 68)
*Mussa x paradisiaca* (Fl 56, Ur 19)
**Muscular/Skeletal**
*Epiphyllum sp*. (Fl 100, Ur 25)
*Nopalea sp*. (Fl 83, Ur 10)
*Peperomia maculosa* (Fl 95, Ur 104)
**Cultural Conditions**
*Baccharis salicina* (Fl 94, Ur 31)
*Clinopodium brownei* (Fl 89, Ur 23)
*Ocimum basilicum* (Fl 69, Ur 37)
*Ruta graveolens* (Fl 68, Ur 35)
**Other**
*Annona cherimola* (Fl 67, Ur 14)
*Citrus sinensis* (Fl 79, Ur 73)
*Persea americana* (Fl 43, Ur 13)
*Prunus persica* (Fl 72, Ur 21)
**(C) Plants with high use reports (Ur) for all diseases**
*Ageratina ligustrina*, stomach pain (Fl 67, Ur 69)
*Baccharis salicina*, chakiq yaj (Fl 82, Ur 27)
*Catoferia chiapensis*, headache (Fl 70, Ur 109)
*Citrus sinensis*, depression (Fl 79, Ur 73)
*Cymbopogon citratus*, fever (Fl 72, Ur 80)
*Ambrosoides Dysphania*, worming (Fl 85, Ur 75)
*Epiphyllum sp*., breaks and sprains (Fl 92, Ur 23)
*Ocimum basilicum*, chakiq yaj (Fl 56, Ur 30)
*Peperomia maculosa*, arthritis (Fl 22, Ur 24)
*Peperomia maculosa*, cramps (Fl 32, Ur 36)
*Peperomia maculosa*, muscle pain (Fl 31, Ur 34)
*Ruta graveolens*, chakiq yaj (Fl 56, Ur 29)
*Smallanthus maculatus* var. *Maculatus*, wounds (Fl 90, Ur 66)

**Table 4:** Informant consensus factor for the estudied Q'eqchi' communities, by category.

**No**.	**Category**	**References**	**Taxa**	**FIC**
1	Gastrointestinal	432	47	0.89
2	Respiratory	194	35	0.82
3	Pain/Fever	376	44	0.89
4	Dermatology	200	34	0.83
5	Muscular/Skeletal	244	33	0.87
6	Cardiovascular	12	8	0.36
7	Urological	26	11	0.60
8	Diabetes	6	5	0.20
9	Reproductive Health	55	21	0.63
10	Cultural conditions	269	36	0.87
11	Oncology	–	–	–
12	Other	241	47	0.81

### Diseases

In this study, to directly answer the investigation question, we determined that the main diseases within the category of gastrointestinal conditions were diarrhea (Nume'sa'), stomach pain and worms (Luqum). The category of respiratory diseases included flu (Ojb') and cold (Wosol). In the category of pain/fever, headache (rail jolom) and fever (Tiq') were the most important. In the dermatological conditions category, wounds (Yo'q'ol), burns (Q'atal), and blisters were important. Of the muscular/skeletal afflictions, arthritis (Much Quej), cramps (much quej), body pain (much Quej), fractures and sprains were the most relevant. Finally, among other afflictions, Chakiq yaj (dehydration) was the most common. Our interpretation of the correlation between the most common diseases and Q'eqchi concepts is presented in Table 2.

### Plants proposed for further phytochemical and pharmacological studies

After our analysis of the data, we conclude (see below) that the plants presented in this section are good candidates for further pharmacological studies. Here, we present information on each plant as a remedy:

*Ageratina ligustrina* (DC.) R.M. King & H. Rob. (Baq'che', Ka che', Kaq'xik'ai, Xica'ilche', pale and bitter leaf tree): three tender shoots of fresh leaves (4 g) are prepared in a decoction in four cups of water, and a cup is taken 3 times a day. It is used to relieve diarrhea and stomach pain.

#### *Baccharis inamoena* gardner (santo domingo, Tisib)

A decoction of the tender leaves (23 g) in four glasses of water is used to relieve the discomfort of flu and cough (fever, headache, and cough). Three cups a day are drunk.

#### *Baccharis salicina* Torr. & A. gray (chilca)

A decoction of the fresh aerial parts (30 g) is prepared together with other plants in 3 liters of water and left to cool overnight. The decoction is used to relieve Chakiq'yaj (dry sick). The child bathes at 6 a.m., every day, until improvement is noticed.

#### *Calea integrifolia* (DC.) hemsl. (rok sosol, rok acach)

Sage that contains the fresh bark of the stem (4 g) is squeezed. The resulting juice (10-15 drops) is used to stop bleeding and heal wounds.

#### *Catoferia chiapensis* A. gray ex benth. (baq'che, baq'laq che', tree with cob flowers)

This plant is used to treat headache. Fresh leaves (8 g) are placed warm on the forehead as a poultice. Additionally, a decoction of tender leaves (30 g) is boiled in four cups of water and consumed 3 times a day.

#### *Clinopodium brownei* (SW.) kuntze (xa'aw tz'i, dog vomit)

This plant is mainly used to treat Chikiq'Yaj (dry sick). The aerial parts (21 g) are macerated and placed in three liters of water along with other plants and left overnight. The next day, at 6:00 am, the sick child is bathed.

#### *Peperomia maculosa* (L.) hook (par q'een, skunk leaf)

The large and ripe succulent leaves (20 g) are macerated, and a decoction is made in four cups of water; a cup is drunk three times a day. This plant is used to treat inflammatory pain in different parts of the body, the whole leaves are tied warm on the area with pain as a poultice and left until they dry.

#### *Smallanthus maculatus* var. *maculatus* (cav.) H. rob. (Ax, arnica)

The tender leaves are dried (4 g) and then macerated, and the powder is placed on wounds to aid in healing.

## Discussion

In ethnopharmacological field studies, there are two main factors to consider when studying plant use: consensus and variation. Therefore, the analysis of plants used by a specific community should be directed at finding patterns. Medicinal plants are not randomly selected; within a culture, plant use and the main reason for the use of a plant demonstrate specific patterns based on the perception of the effectiveness of the plant within the community (Heinrich et al., 1998).

Plants considered to be culturally important are those used by the highest number of people within a category of traditional use, while plants that are mentioned by only one or two informants are of little cultural value. Informant consensus is a concept that was developed to identify plants that are potentially effective as medicines (Heinrich et al., 1998).

In the present study, there was consensus between informants in the categories of gastrointestinal, respiratory, pain/fever, dermatological, muscular/skeletal, and cultural conditions. These results are obvious because normally, people treat the symptoms rather than the underlying cause of illness, and it is easy to distinguish symptoms, such as diarrhea, cough, pain, skin rash, or weakness attributed to a “bad wind,” between these categories. The ability to distinguish these symptoms can be contrasted with high blood pressure, which is also a symptom with different etiologies but is not easy to discriminate without a method to measure the pressure. In agreement with the informants, we predict that the plants used in these categories with a broad reputation (use reports) are likely to have a pharmacological effect. One of the research questions in the present work was, Do the informants appreciate the use of a species for one illness or symptom? We propose the plants listed in Table 3C for further pharmacological studies because the aim of the present work was to find species that can be tested for a possible mechanism of action.

To determine whether there is a correlation between the use of plants or diseases reported in this study with uses reported in previous studies in the area, we present a comparison between the results of this study and those of previous studies.

When we performed a comparison with the Q'eqchi' of Belize (Treyvaud et al., 2005), we found that the communities in the present study share with the communities of Belize recognition of the importance of medicinal plants to treat gastrointestinal, respiratory, reproductive and muscle/skeletal conditions. For the Q'eqchi' of Guatemala, medicinal plants are also important for pain/fever and skin, psychological and other conditions, while for the Q'eqchi' of Belize, the use of medicinal plants against poisons and animal bites, nutritional disorders, disorders of the nervous system, infections, mental disorders, and disorders of the endocrine system is particularly important. For Q'eqchi' traditional healers (Hitziger et al., 2016), the categories of Central Nervous System & Behavioral Syndromes, Reproductive System and General Tissue Problems & Infections were the most important (Table 5).

**Table 5:** Comparison of the Informant consensus factor between different studies in the area.

**Category**	**A**	**B**	**C**	**D**
	**Ch'orti' (Guate.)**	**Q'eqchi' (Belize)**	**Q'eqchi' (Guate.) Specialist**	**Q'eqchi'(Guate.) Normal Population**
Gastrointestinal	0.69	0.71	0.27	0.89
Pain/fever or headache with fever or fever (includes Malaria)	0.72			0.89
Respiratory	0.65	0.70	0.24	0.82
Skin problems	0.51	0.58	0.38	0.83
Psychological/ Spiritual or cultural affinities	0.60	0.58	0.08	0.87
Oral Health	0.50		
Urogenital	0.39		0.30	0.60
Ears and eyes	0.53		
Women's pair remedies or Gynecologic/andrologico	0.69	0.76	0.38	0.63
Poisons and animal bites		0.76	0.37
Other/not classified				0.81
Skeleton/Muscle		0.72	0.32	0.87
Cardiovascular		0.52	0.11	0.36
Diabetes		0.57		0.20
Nutritional Disorders		0.77	
Disorders of the Nervous System		0.69	0.43
Infections		0.68	
Mental disorders		0.67	
Disorders of the endocrine system		0.82	0.13
Pregnancy/Birth/Puerperium		0.57	
Wounds		0.36	
Sensory Disorders		0.25	

*A. Kufer et al. (2005), B. Treyvaud et al. (2005), C. Hitziger et al. (2016), D. Current Study*.

The Ch'orti' and the Q'eqchi', both in Guatemala, share a recognition of the importance of medicinal plants for the treatment of gastrointestinal diseases, pain/fever, and respiratory, psychological and reproductive illnesses. Furthermore, for the Q'eqchi', medicinal plants are important for the treatment of skin disorders and muscular/skeletal conditions, while for the Ch'orti', conditions of the ears and eyes are important (Kufer et al., 2005).

It is remarkable that the Fic in the category of Digestive System is lower (0.27) among traditional healers (Hitziger et al., 2016) than for the normal population interviewed here (0.89), while for disorders of the Central Nervous System & Behavioral Syndromes, specialists have better consensus (0.43) than the normal population (0.87). This result could indicate that people treat gastrointestinal disorders by themselves rather than visiting a specialist. It is important to highlight that according to a previous work (Treyvaud et al., 2005), cardiovascular diseases and diabetes occupy a place of importance for the Q'eqchi' of Belize, and these conditions were not important for the Q'eqchi' in the present study.

In the present study, the category of gastrointestinal afflictions had the highest consensus, and the people treat illnesses using a mixture of bitter, aromatic and mucilaginous species. The bitter species include *A. ligustrina* (Baq'che), which is used to treat diarrhea and stomach pain; *Musa x paradisiaca* (tul), whose green fruit is used to relieve diarrhea in children; *Polypodium lindenianum* (Tis q'een), whose fronds are used to treat stomach pain; and *Psidium guajava* (Pata), which is used to treat diarrhea. Among the aromatic plants, *Dysphania ambrosioides* (Apasote, Isquiij, or pur) is used against worms. The mucilaginous plants of note are *Plantago australis* and *Aloe vera*, which are used to treat gastritis. Dieseldorff (1977) reported that the Q'eqchi' of Alta Verapaz use *D. ambrosioides* against earthworms and *A. ligustrina* to treat colic and inflammation of the stomach, so our results are in agreement with this previous observation. Among specialists, the main use of *D. ambrosioides* is also for digestive ailments, while an important species in this study, *A. ligustrina*, was not mentioned by specialists (Hitziger et al., 2016).

A study conducted with plants used by the Tzotzil and Tzeltal in the montane forests of Chiapas showed the effect of *A. ligustrina* as an antispasmodic agent. Experiments were performed in the ilea of male guinea pigs and confirmed action at a concentration of 250 mg/mL (Tortoriello et al., 1995).

Within the category of pain/fever, we found that aromatic species such as *Catopheria chiapensis* (Baq'rus), *Cymbopogon citratus* (lemon), *Chojte scaberrima* (Phyla), and *Siparuna thecaphora* (Chu che) were important for relief from headaches and fever; additionally, *Coffea arabica* (coffee) and *B. inamoena* (Tisib, “Santo Domingo”), which lack aroma, were important for the same types of conditions. Within this category, the most important species are *C. chiapensis, C. citratus, B. inamoena*, and *C. arabica* (Tables 3B,C). These species were also the most important species for the treatment of respiratory conditions, which shows us that plants used for the treatment of pain/fever are the same as those used to treat symptoms of common respiratory ailments such as the flu and the common cold. A brief survey of medicinal plants of the Q'eqchi' (Dieseldorff, 1977) mentions *B. inamoena* and *C. citratus* as plants used to treat fevers and *C. chiapensis* as a plant used to treat headaches. Among specialists (Hitziger et al., 2016), *C. chiapensis* is not reported, and *C. citratus* is mainly used to treat blood pressure, in agreement with the results presented here, which also reported its use as an expectorant, while *B. inamoena* is reported for cramps, among other uses.

For muscle/skeletal conditions (0.87), the most important species is *P. maculosa* (Paar q'een), which is a succulent and aromatic used to treat arthritis, cramps, and body pain, followed by *Epiphyllum* sp. (Tiq'leb'baq), which is a succulent, non-aromatic plant that resembles a bony leg and is used to treat fractures and sprains. In a previous study (Dieseldorff, 1977), the authors mention that *Epiphyllum* sp. is used for the treatment of fissured bones, and *P. maculosa* is ingested to treat stomach pain and used as a poultice to treat inflammation. Among specialists, other species of *Peperomia* were reported for ulcers or skin problems, but *P. maculosa* was not mentioned, while *Epiphyllum hookeri* was used to treat fractures (Hitziger et al., 2016); both previous observations agree with our results.

Among cultural conditions (0.87), the disease that defined the most important plants for the treatment of these conditions is called Chaquiq'yaj (a condition in which sick children become dehydrated). The symptoms of the disease include diarrhea, vomiting, fever, lack of appetite, whitish eyes, and thirst, which could be associated with a gastrointestinal illness, specifically rotavirus infection, for which the main symptoms are diarrhea, vomiting, malaise, and fever. Indeed, vomiting is a hallmark of rotavirus infection that contributes to dehydration (Crawford et al., 2017). Based on the same observed symptoms, a correlation between Chaquiq'yaj and rotavirus infection can be inferred; however, to prove this correlation, a portable rotavirus test must be conducted with ill children in the communities to eliminate other possible causes, such as noroviruses, enteric adenoviruses, *Escherichia coli* or *Salmonella*. However, the hallmarks of a rotavirus infection (diarrhea, vomiting and dehydration) are also the hallmarks of Chaquiq'yaj.

For the treatment of Chaquiq'yaj, plants are placed in a container of water and left in the open all night, exposed to the cold of the “serene” (night moisture); then, at 6:00 a.m., the sick child is bathed with this water. The Ch'orti of Guatemala have the same practice, in which they let plants soak outdoors to be enhanced by the power of cold temperature (Kufer et al., 2005). The plants used for the treatment of this condition are aromatic; the most important among them, i.e., the plants most frequently mentioned, are *B. salicina* (chilca), *Ruta graveolens* (ruda), *Ocimum basilicum* (albahaca), and *C. brownei* (Xa'aw Tzi).

Q'eqchi' treatment of the disease seeks to lower fever in a cold-water bath with aromatic species. We could not identify this condition among the data reported for healers (Hitziger et al., 2016, 2017).

Species to treat dermatological conditions, such as *C. integrifolia* (Rok'sosol, Rok'acach) and *S. maculatus* var. *maculatus* (Ax), were the most important in terms of use (see Tables 3B,C); the red-brown juice of *Smallanthus*, which resembles blood, is found in the epidermis of the stems and is used to stop the bleeding of wounds. Juice and mucilage from other species are also used; for example, the sap from the stems of *Musa x paradisiaca* (Manzano, tulle) and the mucilage from the fruits of *Sechium edule* (guisquil) are used to heal blisters and burns. *Salmea scandens* is an important species used to treat skin disorders such as itchiness and granular itching. Finally, the aerial parts and the root of *Sida rhombifolia* are used for the treatment of alopecia. Among healers, *Calea* sp. is used to treat ulcers, while *S. maculatus* is not mentioned (Hitziger et al., 2016).

In the category of “other conditions,” *Annona cherimola, Citrus sinensis*, and *Prunus persica* are species of importance because they were mentioned for their common use for the treatment of depression, i.e., when a person wants to ameliorate sadness, anger, or anxiety. Traditionally, these species are used in infusions. In addition, *Persea americana*, whose seeds are used as abortifacients and to treat mumps, is important in this category. In contrast to specialists, who reported the use of *Erythrina berteroana, Tilia platiphyllus*, and *Verbena litoralis* for central nervous system and behavioral syndromes, we detected the use of very common species to treat these illnesses.

It is well known that diabetes and cardiovascular problems are public health concerns worldwide. In this regard, the informants from the Q'eqchi' communities studied here showed that these conditions had low consensus, with a Fic value of 0.20 for diabetes and 0.36 for cardiovascular conditions (Table 4). Throughout this study, only two cases of diabetes were documented, both in the community of Chirrepec; in the rest of communities, the families interviewed did not mention suffering from these diseases.

Exercise from daily walks and constant work together with a diet low in fat and rich in vegetables is an explanation for the low rates of occurrence of these diseases. Either way, the low number of use reports shows that in the popular consciousness of the Q'eqchi' communities studied, there is no experience in the use of medicinal plants to treat diabetes and cardiovascular diseases.

## Conclusions

The categories of disease with the highest values of consensus include gastrointestinal, respiratory, pain/fever, dermatological, muscular/skeletal, and cultural conditions. The results reflect the treatment of symptoms of diarrhea, cough, pain, and skin rashes, according to emic perception. This context means that the informants treat the diarrhea complex, regardless of the etiology.

We observe a high agreement of consensus in the gastrointestinal category. Thus, we can conclude that people trust the use of *A. ligustrina* and *D. ambrosioides*. It is remarkable that in previous studies among specialists, *Ageratina* was not mentioned, which indicates that different results can be obtained when specialists or laypeople are interviewed. However, they share the use of *D. ambrosioides*. Previous observations are reinforced when respiratory conditions and pain/fever are analyzed. In the present study, an important plant for treating these conditions was *C. chiapensis*, the use of which was not reported among the specialists (Hitziger et al., 2016).

Lower consensus regarding plant use in the gastrointestinal category was found among specialists relative to the high consensus found here. This outcome can be explained because we primarily interviewed housewives and farmers who use medicinal plants to resolve day-to-day health problems. In contrast, the specialists shared better agreement regarding the use of plants for the treatment of psychiatric conditions. This agreement may mean that non-specialists attempt to treat their health issues by themselves and visit a specialist for the treatment of more complex situations.

In the present study, we suggest that the cultural condition called Chaquiq'yaj may be correlated with rotavirus infection (see above; cultural conditions), but further studies are necessary to prove this hypothesis. Cooperative research, as proposed previously (Hitziger et al., 2016), may aid in understanding the disease and proposing new treatments.

It is remarkable that conditions such as obesity, hypertension and diabetes are not yet a problem in the studied communities. This outcome can be correlated with daily physical activity, such as walking, and with diet, which is free of processed foods. When we performed interviews, we observed two important conditions. First, the lack of public transportation forces the informants to walk for 1 to 2 h per day. Moreover, all daily activities are carried out on foot. Second, we did not see widely available “miscellaneous” food items, meaning that no soda or processed foods are immediately available. Obtaining a soda requires at least a 1-h walk. This fact certainly contributes to the low levels of cardiovascular diseases or diabetes. It will be of interest to follow such populations to observe them as their lifestyle changes, such as when they no longer need to walk long distances or when sodas are introduced.

Based on the results presented here, we can conclude that *A. ligustrina, C. chiapensis, B. inamoena, P. maculosa, B. salicina, C. brownei, C. integrifolia*, and *S. maculatus* var. *maculatus* are the most culturally relevant species. Because pharmacological studies of these species are not reported in the international literature, further studies are needed to assess the activity of these plants, and phytochemical studies are needed to determine their composition.

## Author contributions

AA-C envisioned the study, wrote the manuscript, and obtained the financial support. JV perform the field study.

### Conflict of interest statement

The authors declare that the research was conducted in the absence of any commercial or financial relationships that could be construed as a potential conflict of interest.

## Abbreviations

F_IC_: Informant consensus factor
Fl: fidelity level
Um: Use-mentions.
