# Supplementary material for: Ethnopharmacological Field Study of Three Q'eqchi Communities in Guatemala
Source: Front Pharmacol. 2018 Nov 6;9:1246. doi: 10.3389/fphar.2018.01246 (PMC6240767; doi:10.3389/fphar.2018.01246)
Supplement: Supplementary file 1 [file Table_1.DOCX]

Table 1. List of species, voucher number, form of use, application form, Friedman fidelity index and Use reports for the medicinal species of the communities of Sanimtaq'a, Santo Domingo las Cuevas and Chirrepec.

| **Scientific Name** | **Voucher** | **Name** | **Form** | **Used Part** | **Diseases** | **Preparation** | **Application** | **Cat** | **Ur** | **Fl** |
| --- | --- | --- | --- | --- | --- | --- | --- | --- | --- | --- |
| *Abelmoschus moschatus* Medik.  (Malvaceae) | 1409 | Alguacil, May xul | Sh | Lv  10-15 g | Cramp | Po | Topic | 5 | 1 | 17 |
|  |  |  |  |  | Muscle Pain | Po | Topic | 5 | 1 | 33 |
|  |  |  |  |  | Stomach ache | Inf | Oral | 1 | 1 | 50 |
|  |  |  |  | Se  1-2 g | Muscle Pain | Ma | Oral | 5 | 1 |  |
|  |  |  |  |  | Stomach ache | De | Oral | 1 | 1 |  |
|  |  |  |  |  |  | Ma | Oral | 1 | 1 |  |
| *Acalypha aristata* Kunth.  (Euphorbiaceae) | 1393 | Hierba del cáncer | He | Lv  3 g | Stomach ache | De | Oral | 1 | 1 | 100 |
| *Achillea millefolium* L.  (Compositae) | 1447 | Curarina | He | Lv  3 g | Depression | De | Oral | 12 | 1 | 100 |
| *Ageratina ligustrina* (DC.) R.M. King & H. Rob.  (Compositae) | 1406 | Baq´che´, Kaq´xik´ai, Xika´ilche´ (árbol de hoja pálida y amarga), Kache | Tr | Lv  4 g | Chakiq yaj | De | Oral | 10 | 1 | 1 |
|  |  |  |  |  | Diarrhoea | De | Oral | 1 | 22 | 21 |
|  |  |  |  |  | Dyspepsia | De | Oral | 1 | 1 | 1 |
|  |  |  |  |  | Gastritis | De | Oral | 1 | 8 |  |
|  |  |  |  |  |  |  | Chew | 1 | 1 | 9 |
|  |  |  |  |  | Itch | De | Bath | 4 | 1 | 1 |
|  |  |  |  |  | Stomach ache | De | Oral | 1 | 69 | 67 |
| *Ageratum conyzoides* (L.) L.  (Compositae) | 1480 | Lo' Q'am, Mejorana | He | Br 10 g | Rupture, sprain | Po | Topic | 5 | 1 | 8 |
|  |  |  |  | Lv  4-6 g | Cough | De | Oral | 2 | 2 | 25 |
|  |  |  |  |  | Fever | De | Oral | 3 | 3 | 25 |
|  |  |  |  |  | Flu | De | Oral | 2 | 2 | 17 |
|  |  |  |  |  | Headache | De | Bath | 3 | 1 |  |
|  |  |  |  |  |  |  | Oral | 3 | 1 | 17 |
|  |  |  |  | Rt 4 g | Cough | De | Oral | 2 | 1 |  |
|  |  |  |  | St 10 g | Wounds | Po | Topic | 4 | 1 | 8 |
| *Allium cepa* L.  (Amaryllidaceae) |  | Cebolla | He | Cat | Flu | De | Oral | 2 | 1 | 50 |
|  |  |  |  |  | Menstruation | D | Oral | 9 | 1 | 50 |
| *Allium sativum* L.  (Amaryllidaceae) |  | Ajo, Anx, Jolom q´een (hoja con cabeza) | He | Cat | Blood pressure | N.A | Oral | 6 | 1 | 2 |
|  |  |  |  |  | Chakiq yaj | D | Bath | 10 | 1 |  |
|  |  |  |  |  |  | So | Bath | 10 | 1 |  |
|  |  |  |  |  |  |  | Oral | 10 | 1 | 6 |
|  |  |  |  |  | Cough | De | Oral | 2 | 3 | 8 |
|  |  |  |  |  | Diarrhoea | De | Oral | 1 | 1 |  |
|  |  |  |  |  |  |  | Oral | 1 | 2 | 6 |
|  |  |  |  |  | Dyspepsia | De | Oral | 1 | 1 | 2 |
|  |  |  |  |  | Fever | De | Oral | 3 | 1 | 4 |
|  |  |  |  |  | Flu | De | Oral | 2 | 1 | 2 |
|  |  |  |  |  | Granos | Po | Topic | 4 | 1 | 2 |
|  |  |  |  |  | Menstruation | De | Oral | 9 | 1 | 2 |
|  |  |  |  |  | Mu | Bu | Inhalated | 10 | 15 | 29 |
|  |  |  |  |  | PMS | De | Oral | 9 | 1 | 2 |
|  |  |  |  |  | Rotavirus | Bu | Inhalated | 12 | 1 | 2 |
|  |  |  |  |  | Scare | Bu | Inhalated | 10 | 1 | 2 |
|  |  |  |  |  | Stomach ache | De | Oral | 1 | 2 |  |
|  |  |  |  |  |  | Fr | Oral | 1 | 1 |  |
|  |  |  |  |  |  | Inf | Oral | 1 | 1 |  |
|  |  |  |  |  |  | Ma | Oral | 1 | 2 |  |
|  |  |  |  |  |  |  | Oral | 1 | 2 | 16 |
|  |  |  |  |  | Throat infection | Inf | Oral | 2 | 1 | 2 |
|  |  |  |  |  | Vermifuge | De | Oral | 1 | 3 |  |
|  |  |  |  |  |  | Inf | Oral | 1 | 1 |  |
|  |  |  |  |  |  | Ma | Topic | 1 | 1 |  |
|  |  |  |  |  |  |  | Topic | 1 | 1 | 12 |
|  |  |  |  |  | Weakness | Bu | Inhalated | 5 | 1 | 2 |
|  |  |  |  | Fr | Cough | De | Oral | 2 | 1 |  |
|  |  |  |  | Lv | Fever | De | Oral | 3 | 1 |  |
| *Aloe vera* (L.) Burm.  (Asparagaceae) |  | Sábila | He | Lv | Alopecia | Po | Topic | 4 | 1 |  |
|  |  |  |  |  |  |  | Topic | 4 | 3 | 22 |
|  |  |  |  |  | Arthritis | Po | Topic | 5 | 1 | 6 |
|  |  |  |  |  | Burns, blister |  | Topic | 4 | 1 | 6 |
|  |  |  |  |  | Gastritis | N.A | Oral | 1 | 4 |  |
|  |  |  |  |  |  |  | Oral | 1 | 6 | 56 |
|  |  |  |  |  | Hepatitis |  | Oral | 12 | 1 | 6 |
|  |  |  |  |  | Vermifuge | De | Oral | 1 | 1 | 6 |
| *Ambrosia peruviana* Willd.  (Asteraceae) | 1459 | Kaki ax. Kaki pim | He | Lv  30 g | Blocked ear | Po | Topic | 12 | 1 | 9 |
|  |  |  |  |  | Chakiq yaj | De | Bath | 10 | 3 | 91 |
|  |  |  |  |  |  | Ma | Bath | 10 | 1 |  |
|  |  |  |  |  |  |  | Oral | 10 | 1 |  |
|  |  |  |  | Lv, st  30 g | Chakiq yaj | De | Bath | 10 | 1 |  |
|  |  |  |  |  |  | Inf | Bath | 10 | 1 |  |
|  |  |  |  |  |  | So | Bath | 10 | 2 |  |
|  |  |  |  | Rt 10 g | Chakiq yaj | De | Bath | 10 | 1 |  |
| *Annona cherimola* Mill.  (Annonaceae) | 1318 | Anona, Tzumuy | Tr | Lv  16 g | Depression | De | Oral | 12 | 13 | 67 |
|  |  |  |  |  |  | Inf | Oral | 12 | 1 |  |
|  |  |  |  |  | Dyspepsia | De | Oral | 1 | 1 | 5 |
|  |  |  |  |  | Gastritis | De | Oral | 1 | 1 | 5 |
|  |  |  |  |  | Headache | De | Oral | 3 | 1 | 5 |
|  |  |  |  |  | Itch | De | Bath | 4 | 1 | 5 |
|  |  |  |  |  | Muscle Pain after Child Birth | De | Bath | 5 | 1 | 5 |
|  |  |  |  |  | Rupture, sprain | Po | Topic | 5 | 1 | 5 |
|  |  |  |  |  | Stomach ache | De | Oral | 1 | 1 | 5 |
| *Anthurium pentaphyllum* var. *bombacifolium* (Schott.) Madison  (Araceae) | 1350 | Manitas | Ep | Lv  16 g | Madness | De | Bath | 12 | 1 |  |
|  |  |  |  |  |  |  | Oral | 12 | 1 | 100 |
| *Arthrostemma ciliatum* Pav. ex D. Don  (Melastomataceae) | 1312 | Chamajij, chamajil, caña de rayo, ruq salkaaq (ruq: caña; sa´ li: del; kaaq: rayo) | Sh | St  20 g | heartburn, acidity | Ch | Oral | 1 | 1 | 11 |
|  |  |  |  |  | Urinary infection | De | Oral | 7 | 6 |  |
|  |  |  |  |  |  |  | Chew | 7 | 1 |  |
|  |  |  |  |  |  |  | Oral | 7 | 1 | 89 |
| *Baccharis inamoena* Gardner  (Compsitae) | 1344 | Santo Domingo, Tisib | Sh | Lv  23 g | Chakiq yaj | Ma | Bath | 10 | 1 |  |
|  |  |  |  |  |  |  | Oral | 10 | 1 | 3 |
|  |  |  |  |  | Cough | De | Oral | 2 | 7 | 12 |
|  |  |  |  |  | Depression | De | Oral | 12 | 6 | 10 |
|  |  |  |  |  | Fever | De | Bath | 3 | 2 |  |
|  |  |  |  |  |  |  | Oral | 3 | 7 |  |
|  |  |  |  |  |  | Po | Topic | 3 | 1 | 17 |
|  |  |  |  |  | Flu | De | Oral | 2 | 13 | 22 |
|  |  |  |  |  | Gastritis | De | Oral | 1 | 1 |  |
|  |  |  |  |  |  | Inf | Oral | 1 | 1 | 3 |
|  |  |  |  |  | Headache | De | Bath | 3 | 2 | 15 |
|  |  |  |  |  |  |  | Oral | 3 | 5 |  |
|  |  |  |  |  |  | Inf | Oral | 3 | 1 |  |
|  |  |  |  |  |  | Po | Topic | 3 | 1 |  |
|  |  |  |  |  | Muscle Pain | De | Bath | 5 | 2 |  |
|  |  |  |  |  |  |  | Oral | 5 | 1 | 5 |
|  |  |  |  |  | Muscle Pain after Child Birth | De | Bath | 5 | 2 |  |
|  |  |  |  |  |  | Inf | Bath | 5 | 1 | 5 |
|  |  |  |  |  | Scare | De | Oral | 10 | 1 | 2 |
|  |  |  |  |  | Stomach ache | De | Oral | 1 | 4 | 7 |
| *Baccharis salicina*Torr. & A.Gray  (Compositae) | 1413 | Chilca | Sh | Lv  30 g | Chakiq yaj | De | Bath | 10 | 17 |  |
|  |  |  |  |  |  |  | Oral | 10 | 3 |  |
|  |  |  |  |  |  | Inf | Bath | 10 | 3 |  |
|  |  |  |  |  |  | So | Bath | 10 | 3 |  |
|  |  |  |  |  |  |  | Oral | 10 | 1 | 82 |
|  |  |  |  |  | Depression | De | Bath | 12 | 1 |  |
|  |  |  |  |  |  |  | Oral | 12 | 1 | 6 |
|  |  |  |  |  | Mal de ojo | De | Bath | 10 | 1 |  |
|  |  |  |  |  |  | N.A | Topic | 10 | 1 |  |
|  |  |  |  |  |  |  | Topic | 10 | 2 | 12 |
| *Bidens alba* var. *radiata* (Sch. Bip) R.E. Ballard  (Compositae) | 1292 | Aceitillo | He | Lv  9 g | Diabetes | De | Oral | 8 | 1 | 100 |
| *Bocconia gracilis* Hutch  (Papaveraceae) | 1470 |  | Tr | Latex  2-3 drops | Toothache |  | Oral | 3 | 1 | 50 |
|  |  |  |  | Lv  4-6 g | Hepatitis | De | Oral | 12 | 1 | 50 |
| *Bougainvillea glabra* Choisy  (Nyctaginaceae) | 1507 |  | Sh | Fl  9 g | Cough | De | Oral | 2 | 1 | 33 |
|  |  |  |  |  | Fever | De | Oral | 3 | 1 | 33 |
|  |  |  |  |  | Flu | Inf | Oral | 2 | 1 | 33 |
| *Byrsonima crassifolia* (L.) Kunth  (Malpighiaceae) | 1500 | Nance | Tr | Br  20 g | Diarrhoea | De | Oral | 1 | 1 | 25 |
|  |  |  |  |  | Dysentery | De | Oral | 1 | 2 | 50 |
|  |  |  |  | Lv  10 g | Toothache | De | Oral | 3 | 1 | 25 |
| *Calea integrifolia* (DC.) Hemsl.  (Compositae) | 1466 | Rok Sosol, Rok acach | Sh | Lv  4-6 g | Wounds | Po | Topic | 4 | 1 |  |
|  |  |  |  | St, juice  15 drops | Wounds | Po | Topic | 4 | 20 | 100 |
| *Camellia sinensis* (L.) Kuntze  (Theaceae) | 1535 | Té | Tr | Lv  7 g | Anxiolytic | De | Oral | 12 | 1 | 6 |
|  |  |  |  |  | Blood cleaner | De | Oral | 6 | 1 |  |
|  |  |  |  |  |  | Inf | Oral | 6 | 1 | 12 |
|  |  |  |  |  | Diabetes | De | Oral | 8 | 1 | 6 |
|  |  |  |  |  | Diuretic | De | Oral | 7 | 1 | 6 |
|  |  |  |  |  | Gastritis | De | Oral | 1 | 4 |  |
|  |  |  |  |  |  | Inf | Oral | 1 | 2 |  |
|  |  |  |  |  |  |  | Chew | 1 | 2 |  |
|  |  |  |  |  |  |  | Oral | 1 | 1 | 53 |
|  |  |  |  |  | Granos | De | Bath | 4 | 1 | 6 |
|  |  |  |  |  | Lose weight | De | Oral | 12 | 1 | 6 |
|  |  |  |  |  | Stomach ache |  | Chew | 1 | 1 | 6 |
| *Campyloneurum* sp.  (Polypodiaceae) | 1503 | Helecho lengua | Ep | Lv  30 g | Inflammation | Inf | Bath | 5 | 1 | 100 |
| *Catoferia chiapensis* A. Gray ex Benth.  (Lamiaceae) | 1298 | B´aqlaq che´ (Arbol con flores de mazorca), Baqché | Tr | Lv  30 g | Chakiq yaj | De | Bath | 10 | 1 |  |
|  |  |  |  |  |  | Inf | Bath | 10 | 1 |  |
|  |  |  |  |  |  | Ma | Bath | 10 | 1 |  |
|  |  |  |  |  |  |  | Oral | 10 | 1 | 3 |
|  |  |  |  |  | Cough | De | Inhalated | 2 | 1 |  |
|  |  |  |  |  |  |  | Oral | 2 | 4 | 3 |
|  |  |  |  |  | Fever | De | Bath | 3 | 1 |  |
|  |  |  |  |  |  |  | Oral | 3 | 11 |  |
|  |  |  |  |  |  | Po | Topic | 3 | 1 | 8 |
|  |  |  |  |  | Flu | De | Bath | 2 | 1 |  |
|  |  |  |  |  |  |  | Inhalated | 2 | 1 |  |
|  |  |  |  |  |  |  | Oral | 2 | 7 |  |
|  |  |  |  |  |  | Po | Topic | 2 | 1 |  |
|  |  |  |  |  |  |  | Topic | 2 | 1 | 7 |
|  |  |  |  |  | Headache | De | Bath | 3 | 3 |  |
|  |  |  |  |  |  |  | Oral | 3 | 13 |  |
|  |  |  |  |  |  | Po | Topic | 3 | 93 | 70 |
|  |  |  |  |  | Inflammation | De | Bath | 5 | 1 |  |
|  |  |  |  |  |  |  | Oral | 5 | 1 | 1 |
|  |  |  |  |  | Muscle Pain | Alc | Oral | 5 | 1 |  |
|  |  |  |  |  |  | De | Bath | 5 | 5 | 4 |
|  |  |  |  |  | Muscle Pain after Child Birth | De | Bath | 5 | 1 |  |
|  |  |  |  |  |  | Inf | Bath | 5 | 1 | 1 |
|  |  |  |  |  | Weakness | De | Bath | 5 | 1 |  |
|  |  |  |  |  |  |  | Oral | 5 | 1 |  |
|  |  |  |  |  |  | Inf | Bath | 5 | 1 | 2 |
| *Cecropia obtusifolia* Bertol.  (Urticaceae) | 1306 | Guarumo, Choop rojo, Choop blanco | Tr | Lv  30 g | Cough | De | Oral | 2 | 4 | 50 |
|  |  |  |  |  | Cramp | De | Oral | 5 | 1 | 13 |
|  |  |  |  |  | Diabetes | De | Oral | 8 | 1 | 13 |
|  |  |  |  |  | Flu | De | Oral | 2 | 1 | 13 |
|  |  |  |  | St  10 g | Urinary infection | De | Oral | 7 | 1 | 13 |
| *Cinchona officinalis* L.  (Rubiaceae) | 1474 | Quina | Tr | Br  4 g | Dysentery | De | Oral | 1 | 1 | 25 |
|  |  |  |  |  | Gastritis | De | Oral | 1 | 2 | 50 |
|  |  |  |  |  | Toothache | De | Oral | 3 | 1 | 25 |
| *Cinnamomum verum* J. Presl  (Lauraceae) |  | Canela | Tr | Br  4-5 g | Cough | De | Oral | 2 | 6 | 55 |
|  |  |  |  |  | Dyspepsia | De | Oral | 1 | 2 | 18 |
|  |  |  |  |  | Fever | De | Oral | 3 | 1 | 9 |
|  |  |  |  |  | Flu | De | Oral | 2 | 1 | 9 |
|  |  |  |  |  | Urinary infection | Inf | Oral | 7 | 1 | 9 |
| *Cirsium* sp.  (Compositae) | 1326 | Cardosanto | He | Lv  15 g | Chakiq yaj | Ma | Bath | 10 | 1 |  |
|  |  |  |  |  |  |  | Oral | 10 | 1 | 100 |
| *Citrus aurantiifolia* (Christm.) Swingle  (Rutaceae) |  | Limón, lamunx | Tr | Fr | asthma | De | Bath | 2 | 1 |  |
|  |  |  |  |  |  |  | Inhalated | 2 | 1 |  |
|  |  |  |  |  |  |  | Oral | 2 | 1 | 9 |
|  |  |  |  |  | Cough | De | Oral | 2 | 5 |  |
|  |  |  |  |  |  | Inf | Oral | 2 | 1 | 26 |
|  |  |  |  |  | Diarrhoea |  | Oral | 1 | 1 | 3 |
|  |  |  |  |  | Fever | De | Oral | 3 | 1 | 17 |
|  |  |  |  |  | Flu | De | Oral | 2 | 1 | 3 |
|  |  |  |  |  | Headache | De | Bath | 3 | 1 |  |
|  |  |  |  |  |  |  | Oral | 3 | 1 |  |
|  |  |  |  |  |  | Po | Topic | 3 | 2 | 17 |
|  |  |  |  |  | Itch | Po | Topic | 4 | 1 |  |
|  |  |  |  |  |  |  | Topic | 4 | 1 | 6 |
|  |  |  |  | Fr | Dyspepsia | Pre | Oral | 1 | 1 | 3 |
|  |  |  |  |  | Fever | De | Oral | 3 | 2 |  |
|  |  |  |  |  | Stomach ache | Pre | Oral | 1 | 1 | 3 |
|  |  |  |  | Lv  9 g | Cough | De | Bath | 2 | 1 |  |
|  |  |  |  |  |  |  | Oral | 2 | 2 |  |
|  |  |  |  |  | Depression | De | Oral | 12 | 5 | 14 |
|  |  |  |  |  | Fever | De | Oral | 3 | 3 |  |
|  |  |  |  |  | Headache | De | Oral | 3 | 2 |  |
| *Citrus reticulata* Blanco.  (Rutaceae) | 1385 | Mandarina | Tr | Lv  9 g | Depression | De | Oral | 12 | 6 | 100 |
| *Citrus sinensis* (L.) Osbeck  (Rutaceae) |  | Naranja, Chiin | Tr | Fr | Depression | Pre | Oral | 12 | 1 | 79 |
|  |  |  |  | Lv  9 g | asthma | De | Bath | 2 | 1 |  |
|  |  |  |  |  |  |  | Inhalated | 2 | 1 |  |
|  |  |  |  |  |  |  | Oral | 2 | 1 | 3 |
|  |  |  |  |  | Cough | De | Bath | 2 | 1 |  |
|  |  |  |  |  |  |  | Oral | 2 | 2 | 3 |
|  |  |  |  |  | Depression | De | Bath | 12 | 1 |  |
|  |  |  |  |  |  |  | Oral | 12 | 68 |  |
|  |  |  |  |  |  | Inf | Oral | 12 | 1 |  |
|  |  |  |  |  |  | Inf | Oral | 12 | 2 |  |
|  |  |  |  |  | Fever | De | Oral | 3 | 7 | 8 |
|  |  |  |  |  | Flu | De | Oral | 2 | 3 | 3 |
|  |  |  |  |  | Headache | De | Oral | 3 | 1 | 1 |
|  |  |  |  |  | Muscle Pain after Child Birth | De | Bath | 5 | 1 |  |
|  |  |  |  |  |  | Inf | Bath | 5 | 1 | 2 |
| *Citrus* sp.  (Rutaceae) | 1332 | Lima | Tr | Lv  9 g | Depression | De | Bath | 12 | 1 |  |
|  |  |  |  |  |  |  | Oral | 12 | 3 | 100 |
| *Clinopodium brownei* (Sw.) Kuntze  (Lamiaceae) | 1282 | Xa´aw tz´i (vómito de perro) | He | Ap  21 g | Chakiq yaj | De | Bath | 10 | 10 |  |
|  |  |  |  |  |  |  | Oral | 10 | 1 |  |
|  |  |  |  |  |  | Inf | Bath | 10 | 2 |  |
|  |  |  |  |  |  | Ma | Bath | 10 | 2 |  |
|  |  |  |  |  |  |  | Oral | 10 | 2 |  |
|  |  |  |  |  |  | So | Bath | 10 | 3 |  |
|  |  |  |  |  |  |  | Oral | 10 | 2 | 85 |
|  |  |  |  |  | Cough | De | Oral | 2 | 1 | 4 |
|  |  |  |  |  | Fever | Ma | Bath | 3 | 1 |  |
|  |  |  |  |  |  |  | Oral | 3 | 1 | 8 |
|  |  |  |  |  | Scare | De | Bath | 10 | 1 | 4 |
| *Coffea arabica* L.  (Rubiaceae) | 1320 | Café, kape | Tr | Fr  5 g | Fever | De | Oral | 3 | 1 | 28 |
|  |  |  |  | Lv  4 g | Cough | De | Oral | 2 | 3 | 7 |
|  |  |  |  |  | Depression | De | Oral | 12 | 11 | 24 |
|  |  |  |  |  | Dermatitis | Po | Topic | 4 | 1 | 2 |
|  |  |  |  |  | Dyspepsia | De | Oral | 1 | 1 | 2 |
|  |  |  |  |  | Fever | De | Bath | 3 | 1 |  |
|  |  |  |  |  |  |  | Oral | 3 | 11 |  |
|  |  |  |  |  | Flu | De | Oral | 2 | 7 | 15 |
|  |  |  |  |  | Headache | De | Oral | 3 | 4 |  |
|  |  |  |  |  |  | Po | Topic | 3 | 3 | 15 |
|  |  |  |  |  | Muscle Pain | De | Bath | 5 | 1 |  |
|  |  |  |  |  |  |  | Oral | 5 | 1 | 4 |
|  |  |  |  | Rt  6 g | Urinary infection | De | Oral | 7 | 1 | 2 |
| *Cornutia pyramidata* L.  (Lamiaceae) | 1562 | Arbol de la vida, jorob´te´, Tzac asumché | Tr | Lv  7.5 g | Blood cleaner | De | Oral | 6 | 1 | 20 |
|  |  |  |  |  | Blood pressure | De | Oral | 6 | 1 | 20 |
|  |  |  |  |  | Stomach ache | De | Oral | 1 | 3 | 60 |
| *Costus ruber* Griseb.  (Costaceae) | 1445 | K'u (hoja de chucho) | He | Lv  15 g | Chakiq yaj | De | Bath | 10 | 1 | 100 |
| *Costus* sp.  (Costaceae) | 1402 | K'u | He | Lv  15 g | Chakiq yaj | De | Bath | 10 | 1 |  |
|  |  |  |  |  |  | Ma | Bath | 10 | 1 |  |
|  |  |  |  |  |  |  | Oral | 10 | 1 | 100 |
| *Cupressus lusitanica* Mill.  (Cupressaceae) | 1359 | Ciprés | Tr | Br  10 g | Toothache | De | Oral | 3 | 1 | 46 |
|  |  |  |  |  | Weakness | Inf | Bath | 5 | 1 | 8 |
|  |  |  |  | Fr  5 g | Toothache | De | Oral | 3 | 3 |  |
|  |  |  |  |  |  |  | Chew | 3 | 1 |  |
|  |  |  |  | Lv  6 g | Cough | De | Oral | 2 | 1 | 8 |
|  |  |  |  |  | Fever |  | Topic | 3 | 1 | 8 |
|  |  |  |  |  | Inflammation | De | Bath | 5 | 1 |  |
|  |  |  |  |  |  |  | Oral | 5 | 1 | 15 |
|  |  |  |  |  | Muscle Pain after Child Birth | Inf | Bath | 5 | 1 | 8 |
|  |  |  |  |  | sore throat | De | Oral | 2 | 1 | 8 |
|  |  |  |  |  | Toothache | De | Oral | 3 | 1 |  |
| *Cymbopogon citratus* (DC.) Stapf.  (Poaceae) | 1290 | Té de limón, Telemón, k´isk´im | He | Lv  10 g | Cough | De | Oral | 2 | 8 | 8 |
|  |  |  |  |  | Depression | De | Oral | 12 | 1 | 1 |
|  |  |  |  |  | Fever | De | Bath | 3 | 1 |  |
|  |  |  |  |  |  |  | Oral | 3 | 78 |  |
|  |  |  |  |  |  | Po | Topic | 3 | 1 | 72 |
|  |  |  |  |  | Flu | De | Bath | 2 | 1 |  |
|  |  |  |  |  |  |  | Oral | 2 | 14 | 14 |
|  |  |  |  |  | Headache | De | Inhalated | 3 | 1 |  |
|  |  |  |  |  |  |  | Oral | 3 | 5 | 5 |
|  |  |  |  | Rt | Cough | De | Oral | 2 | 1 |  |
| *Datura × candida* (Pers.) Saff.  (Solanaceae) | 1351 | Campanilla | Tr | Lv | Insomnia | N.A | Inhalation | 12 | 1 | 50 |
|  |  |  |  |  | Mumps | Po | Topic | 12 | 1 | 50 |
| Desconocida  (Bignoniaceae) |  | Pens k´aam | Vi | St | Diuretic | De | Oral | 7 | 1 | 100 |
| Desconocida |  | K´aam Yaj | He | Lv | Cramp | De | Oral | 5 | 1 | 100 |
| *Desmodium* sp.  (Leguminosae) | 1293 | Chupil q´een | He | Ap  10 g | Dermatitis | Ma | Topic | 4 | 3 |  |
|  |  |  |  |  |  |  | Topic | 4 | 1 | 100 |
| *Dichaea squarrosa* Lindl  (Orchidaceae) | 1530 | Cienpies | Ep | Lv  10 g | Dermatitis | Ma | Topic | 4 | 1 | 100 |
| *Dysphania ambrosoides* (L.) Mosyakin & Clemants  (Amaranthaceae) | 1277 | Apazote, Isquiij pur | He | Lv  1.2 -2.6 g | Amoebas | De | Oral | 1 | 1 | 1 |
|  |  |  |  |  | Antihemorrhagic | De | Oral | 12 | 2 | 3 |
|  |  |  |  |  | Internal wounds | De | Oral | 12 | 1 | 1 |
|  |  |  |  |  | Metrorrhagia | De | Oral | 9 | 3 | 6 |
|  |  |  |  |  | Scare | De | Bath | 10 | 1 | 1 |
|  |  |  |  |  | Stomach ache | De | Oral | 1 | 1 |  |
|  |  |  |  |  |  | De | Oral | 1 | 1 | 2 |
|  |  |  |  |  | Vermifuge | De | Oral | 1 | 35 |  |
|  |  |  |  |  |  | De | Oral | 1 | 1 |  |
|  |  |  |  |  |  | Inf | Oral | 1 | 2 |  |
|  |  |  |  |  |  | Ma | Topic | 1 | 4 |  |
|  |  |  |  |  |  | N.A | Topic | 1 | 1 |  |
|  |  |  |  |  |  | Po | Topic | 1 | 31 |  |
|  |  |  |  |  |  |  | Topic | 1 | 1 | 85 |
|  |  |  |  | Rt | Antihemorrhagic | De | Oral | 12 | 1 |  |
|  |  |  |  |  | Metrorrhagia | De | Oral | 9 | 2 |  |
|  |  |  |  |  | Vermifuge | De | Oral | 1 | 1 |  |
| *Eleusine* sp.  (Poaceae) | 1412 | Grama, pach´aya´ | He | Lv  12 g | Chakiq yaj | De | Bath | 10 | 1 |  |
|  |  |  |  |  |  |  | Oral | 10 | 1 |  |
|  |  |  |  |  |  | Ma | Bath | 10 | 1 |  |
|  |  |  |  |  |  |  | Oral | 10 | 1 | 100 |
| *Epiphyllum* sp.  (Cactaceae) | 1514 | Tik´leb´b´aq (Junta huesos); Tik´qual b´aq | Sh | St | Cramp | Ma | Topic | 5 | 1 | 4 |
|  |  |  |  |  | Rheumatism | Ma | Topic | 5 | 1 | 4 |
|  |  |  |  |  | Rupture, sprain | Ma | Oral | 5 | 1 |  |
|  |  |  |  |  |  | Po | Topic | 5 | 22 | 92 |
| *Eriobotrya japonica* (Thumb.) Lindl.  (Rosaceae) |  | Níspero | Tr | Lv  20 g | Depression | De | Oral | 12 | 2 | 33 |
|  |  |  |  |  | Gastritis | De | Oral | 1 | 2 | 33 |
|  |  |  |  |  | Headache | Po | Topic | 3 | 1 | 17 |
|  |  |  |  |  | Urinary infection | De | Oral | 7 | 1 | 17 |
| *Eucaliptus* sp.  (Myrtaceae) |  | Eucalipto | Tr | Br | Gastritis | De | Oral | 1 | 1 | 6 |
|  |  |  |  | Lv  8 g | asthma | De | Bath | 2 | 1 |  |
|  |  |  |  |  |  |  | Inhalation | 2 | 1 |  |
|  |  |  |  |  |  |  | Oral | 2 | 1 | 19 |
|  |  |  |  |  | Cough | De | Inhalation | 2 | 2 |  |
|  |  |  |  |  |  |  | Oral | 2 | 3 | 31 |
|  |  |  |  |  | Fever | De | Bath | 3 | 1 |  |
|  |  |  |  |  |  |  | Oral | 3 | 3 | 25 |
|  |  |  |  |  | Flu | De | Inhalation | 2 | 1 |  |
|  |  |  |  |  |  |  | Oral | 2 | 1 | 13 |
|  |  |  |  |  | Headache | De | Inhalation | 3 | 1 | 6 |
| *Eucalyptus cinerea* F.Muell. ex Benth.  (Myrtaceae) |  | Eucalipto | Tr | Lv | Cough | De | Oral | 2 | 1 | 100 |
| *Euphorbia lancifolia* Schltdl.  (Euphorbiaceae) | 1276 | Ixbut | He | Ap  6 g | Galactagogue | De | Oral | 12 | 3 | 100 |
| *Fuchsia parviflora* Lindl.  (Onagraceae) | 1431 | Xcuajsel | Sh | Lv | Chakiq yaj | Inf | Bath | 10 | 1 | 50 |
|  |  |  |  |  | Granos | Ma | Topic | 4 | 1 | 50 |
| *Hydrocotyle mexicana* Schltdl. & Cham.  (Araliaceae) | 1410 | Qis ak | He | Lv  10 g | Chakiq yaj | Ma | Bath | 10 | 1 |  |
|  |  |  |  |  |  |  | Oral | 10 | 1 | 100 |
| *Hyptis atrorubens* Poit.  (Lamiaceae) | 1467 | Xwachupil | He | Lv  5 g | Toothache | De | Oral | 3 | 1 |  |
|  |  |  |  |  |  |  | Oral | 3 | 1 | 100 |
| *Hyptis intermedia* Epling  (Lamiaceae) | 1289 | Katal q´een | He | Lv  5 g | Dermatitis | Ma | Topic | 4 | 1 | 100 |
| *Ipomea batatas* (L.) Lam  (Convolvulaceae) |  | Camote, is | Vi | Lv  10 g | Mumps | Po | Topic | 12 | 1 | 100 |
| *Ipomoea purpurea* (L.) Roth.  (Convolvulaceae) | 1321 | Quiebra cajete, Sa´yub´, sa´tiu | He | Ap | Headache | Po | Topic | 3 | 4 | 100 |
| *Justicia spicigera* Schltdl.  (Acanthaceae) | 1394 | Jiquilite | Sh | Lv  33 g | Chakiq yaj | De | Bath | 10 | 1 |  |
|  |  |  |  |  |  |  | Oral | 10 | 1 |  |
|  |  |  |  |  |  | Ma | Bath | 10 | 1 |  |
|  |  |  |  |  |  |  | Oral | 10 | 3 |  |
|  |  |  |  |  |  | So | Bath | 10 | 1 |  |
|  |  |  |  |  |  |  | Oral | 10 | 1 | 80 |
|  |  |  |  |  | heat/sweat in child | De | Bath | 10 | 2 | 20 |
| *Kohleria spicata* (HBK) Henst.  (Gesneriaceae) | 1335 | Chupil Q'een | He | St | Dermatitis | Ma | Topic | 4 | 1 | 100 |
| *Lantana camara* L.  (Verbenaceae) | 1504 | 5 negritos, cox pim | Sh | Fr  2 g | Cough |  | Chew | 2 | 1 | 100 |
| *Lepidium virginicum* L.  (Brassicaceae) | 1448 | Centavito | He | Ap  30 g | Headache | De | Oral | 3 | 1 | 50 |
|  |  |  |  |  | Madness | De | Oral | 12 | 1 | 50 |
| *Liquidambar styraciflua* L.  (Altingiaceae) | 1316 | Liquidambar, Onc, Okob´ | Tr | Lv  40 g | Antihemorrhagic | De | Oral | 12 | 1 | 6 |
|  |  |  |  |  | asthma | De | Bath | 2 | 1 |  |
|  |  |  |  |  |  |  | Inhalation | 2 | 1 |  |
|  |  |  |  |  |  |  | Oral | 2 | 1 | 17 |
|  |  |  |  |  | Flu | Ma | Topic | 2 | 1 | 6 |
|  |  |  |  |  | Granos | Ma | Topic | 4 | 1 |  |
|  |  |  |  |  |  | Po | Topic | 4 | 1 | 11 |
|  |  |  |  |  | Headache | De | Bath | 3 | 1 | 6 |
|  |  |  |  |  | Inflammation | De | Bath | 5 | 1 | 6 |
|  |  |  |  |  | Itch | De | Bath | 4 | 1 | 6 |
|  |  |  |  |  | Mu | De | Bath | 10 | 1 | 6 |
|  |  |  |  |  | Muscle Pain | De | Bath | 5 | 4 | 22 |
|  |  |  |  |  | Muscle Pain after Child Birth | Inf | Bath | 5 | 1 | 6 |
|  |  |  |  | Re | Cramp | Po | Topic | 5 | 1 | 6 |
|  |  |  |  |  | Rupture, sprain | | Topic | 5 | 1 | 6 |
| *Litzea* sp.  (Lauraceae) |  | Laurel | Tr | Lv  2 g | Cough | De | Oral | 2 | 1 | 100 |
| *Manihot sculenta* Crantz  (Euphorbiaceae) |  | Yuca | Sh | Rt | Urinary infection | Ma | Oral | 7 | 1 | 100 |
| *Matricaria chamomilla* L.  (Compositae) |  | Manzanilla | He | Ap  10 g | Cough | De | Bath | 2 | 1 |  |
|  |  |  |  |  |  |  | Oral | 2 | 2 | 19 |
|  |  |  |  |  | Diarrhoea | De | Oral | 1 | 2 | 13 |
|  |  |  |  |  | Dyspepsia | De | Oral | 1 | 1 | 6 |
|  |  |  |  |  | Menstruation | De | Oral | 9 | 1 |  |
|  |  |  |  |  |  | Inf | Oral | 9 | 1 | 13 |
|  |  |  |  |  | PMS | De | Oral | 9 | 6 | 38 |
|  |  |  |  |  | Stomach ache | De | Oral | 1 | 1 |  |
|  |  |  |  |  |  | Inf | Oral | 1 | 1 | 13 |
| *Mentha spicata* L.  (Lamiaceae) | 1341 | Hierba buena, Isk´i´ij | He | Lv  8 g | asthma | De | Oral | 2 | 1 | 6 |
|  |  |  |  |  | Cough | De | Oral | 2 | 3 | 19 |
|  |  |  |  |  | Depression | De | Oral | 12 | 1 | 6 |
|  |  |  |  |  | Diabetes | De | Oral | 8 | 2 | 13 |
|  |  |  |  |  | Dyspepsia | De | Oral | 1 | 2 |  |
|  |  |  |  |  |  | Inf | Oral | 1 | 1 | 19 |
|  |  |  |  |  | Flu | De | Oral | 2 | 1 | 6 |
|  |  |  |  |  | Gastritis | De | Oral | 1 | 1 | 6 |
|  |  |  |  |  | Stomach ache | De | Oral | 1 | 3 | 19 |
|  |  |  |  |  | Vermifuge | De | Oral | 1 | 1 | 6 |
| *Miconia calvescens* DC.  (Melastomataceae) |  | Xac ché (Catalina) | Tr | Lv  15 g | Blood pressure | De | Oral | 6 | 1 | 100 |
| *Mimosa albida* Willd.  (Leguminosae) | 1288 | Wara K'ix, dormilona espinosa | Sh | Lv  20 g | Anxiolytic |  | Inhalation | 12 | 1 | 33 |
|  |  |  |  |  | Chakiq yaj | Ma | Bath | 10 | 1 |  |
|  |  |  |  |  |  |  | Oral | 10 | 1 | 67 |
| *Monstera adansonii* var. *laniata* (Schott) Madison  (Araceae) | 1463 | Cruz ventana | Ep | Lv | Headache | Po | Topic | 3 | 1 | 50 |
|  |  |  |  |  | Inflammation | De | Bath | 5 | 1 | 50 |
| *Muehlenbeckia platyclados* (F. Muell.) Meisn.  (Polygonaceae) | 1422 |  | Sh | St  15 g | Diarrhoea | De | Oral | 1 | 1 | 25 |
|  |  |  |  |  | Gastritis | De | Oral | 1 | 1 | 25 |
|  |  |  |  |  | Stomach ache | De | Oral | 1 | 2 | 50 |
| *Musa* sp.  (Musaceae) |  | Banano, guineo, guinello tul, manzanotul | Sh | Fr  20 g | Diarrhoea | De | Oral | 1 | 1 |  |
|  |  |  |  |  |  | Ma | Oral | 1 | 10 |  |
|  |  |  |  |  | Gastritis | Ma | Oral | 1 | 1 | 3 |
|  |  |  |  | Sap juice | Burns, blister | N.A | Topic | 4 | 1 |  |
|  |  |  |  |  |  |  | Topic | 4 | 13 | 47 |
|  |  |  |  |  | Heal |  | Topic | 4 | 2 | 6 |
|  |  |  |  |  | Wounds | N.A | Topic | 4 | 1 | 3 |
|  |  |  |  | St | Burns, blister | Po | Topic | 4 | 1 |  |
|  |  | Banano, guineo, guinello tul, manzanotul | Sh | Fr  20 g | Diarrhoea | Ma | Oral | 1 | 2 | 41 |
|  |  |  |  | Sap juice | Burns, blister | N.A | Topic | 4 | 1 |  |
|  |  |  |  |  | Diarrhoea | Ju | Oral | 1 | 1 |  |
| *Myrica cerifera* L.  (Myricaceae) | 1508 | Waut, arrayan | Tr | Lv  25 g | Alopecia | De | Bath | 4 | 1 | 25 |
|  |  |  |  |  | Chakiq yaj | Ma | Bath | 10 | 1 |  |
|  |  |  |  |  |  |  | Oral | 10 | 1 | 50 |
|  |  |  |  |  | Inflammation | De | Bath | 5 | 1 | 25 |
| *Neomarica gracilis* (Herb.) Sprague  (Iridaceae) | 1443 | Q'uq q¡en (hoja cola de Quetzal) | He | Rt  12 g | Menstruation | Inf | Oral | 9 | 1 | 50 |
|  |  |  |  | Se | Granos | De | Bath | 4 | 1 | 50 |
| *Neurolaena lobata* (L.) R. Br. ex Cass.  (Compositae) | 1329 | Tres puntas, ka´woqax, Xka´waqax | He | Lv  3 g | Cough | De | Oral | 2 | 1 | 7 |
|  |  |  |  |  | Diarrhoea | De | Oral | 1 | 2 | 14 |
|  |  |  |  |  | Fever | De | Oral | 3 | 1 | 7 |
|  |  |  |  |  | Gastritis | De | Oral | 1 | 1 | 7 |
|  |  |  |  |  | Manchas | Ma | Topic | 4 | 1 | 7 |
|  |  |  |  |  | Muscle Pain | De | Oral | 5 | 1 | 7 |
|  |  |  |  |  | Stomach ache | De | Oral | 1 | 6 |  |
|  |  |  |  |  |  | Inf | Oral | 1 | 1 | 50 |
| *Nicotiana tabacum* L.  (Solanaceae) | 1280 | Tabaco, May | Sh | Lv  18 g | Chakiq yaj | De | Bath | 10 | 2 |  |
|  |  |  |  |  |  | So | Bath | 10 | 1 | 75 |
|  |  |  |  |  | Mu | Bu | Inhalated | 10 | 1 | 25 |
| *Nopalea cochenilliphera* (L.) Salm Dyck  (Cactaceae) | 1281 | Xab Q'qa Wa, Nopal | Sh | St | Gastritis | N.A | Oral | 1 | 2 | 17 |
|  |  |  |  |  | Rupture, sprain | Po | Topic | 5 | 10 | 83 |
| *Ocimum basilicum* L.  (Lamiaceae) | 1408 | Albahaca | Sh | Lv  42 g | asthma | De | Bath | 2 | 1 |  |
|  |  |  |  |  |  |  | Inhalation | 2 | 1 |  |
|  |  |  |  |  |  |  | Oral | 2 | 1 | 6 |
|  |  |  |  |  | Chakiq yaj | De | Bath | 10 | 1 |  |
|  |  |  |  |  |  | De | Bath | 10 | 14 |  |
|  |  |  |  |  |  |  | Oral | 10 | 4 |  |
|  |  |  |  |  |  | Inf | Bath | 10 | 3 |  |
|  |  |  |  |  |  | Ma | Bath | 10 | 1 |  |
|  |  |  |  |  |  |  | Oral | 10 | 1 |  |
|  |  |  |  |  |  | So | Bath | 10 | 4 |  |
|  |  |  |  |  |  |  | Oral | 10 | 2 | 56 |
|  |  |  |  |  | Depression | De | Oral | 12 | 1 |  |
|  |  |  |  |  |  | De | Oral | 12 | 6 |  |
|  |  |  |  |  |  | Inf | Oral | 12 | 1 | 15 |
|  |  |  |  |  | Fever | De | Oral | 3 | 3 |  |
|  |  |  |  |  |  |  | Topic | 3 | 1 | 7 |
|  |  |  |  |  | Headache | Ma | Oral | 3 | 1 | 2 |
|  |  |  |  |  | heat/sweat in child | Po | Topic | 10 | 1 | 2 |
|  |  |  |  |  | Mal de ojo | De | Bath | 10 | 3 |  |
|  |  |  |  |  |  | N.A | Topic | 10 | 1 |  |
|  |  |  |  |  |  |  | Topic | 10 | 1 | 9 |
|  |  |  |  |  | Mu | Bu | Inhalation | 10 | 1 | 2 |
|  |  |  |  |  | PMS | De | Oral | 9 | 1 | 2 |
| *Oreopanax sanderianus* Hemsl  (Araliaceae) | 1517 |  | Tr | Lv | Scare | Bu | Inhalation | 10 | 1 | 100 |
| *Origanum vulgare* L.  (Lamiaceae) |  | Orégano | Sh | Ap  6 g | Menstruation | Inf | Oral | 9 | 1 | 20 |
|  |  |  |  |  | Muscle Pain after Child Birth | De | Oral | 5 | 1 | 20 |
|  |  |  |  |  | PMS | De | Oral | 9 | 3 | 60 |
| *Paspalum* sp.  (Poaceae) | 1411 | Grama, pach´aya´ | He | Lv  12 g | Chakiq yaj | De | Bath | 10 | 1 |  |
|  |  |  |  |  |  |  | Oral | 10 | 1 |  |
|  |  |  |  |  |  | Ma | Bath | 10 | 1 |  |
|  |  |  |  |  |  |  | Oral | 10 | 1 | 100 |
| *Passiflora ligularis* Juss.  (Passifloraceae) | 1378 | Cramix, granadilla | Vi | Lv  7 g | Gastritis | De | Oral | 1 | 1 | 100 |
| *Peperomia maculosa* (L.) Hook  (Piperaceae) | 1403 | Paar q´een (hoja de zorrillo) | He | Lv  20 g | Arthritis | De | Oral | 5 | 4 |  |
|  |  |  |  |  |  | Po | Topic | 5 | 20 | 22 |
|  |  |  |  |  | Cramp | De | Oral | 5 | 3 |  |
|  |  |  |  |  |  | Ma | Oral | 5 | 1 |  |
|  |  |  |  |  |  | Po | Topic | 5 | 32 | 32 |
|  |  |  |  |  | Headache | Po | Topic | 3 | 5 | 5 |
|  |  |  |  |  | Inflammation | Po | Topic | 5 | 2 | 2 |
|  |  |  |  |  | Muscle Pain | De | Oral | 5 | 5 |  |
|  |  |  |  |  |  | Ma | Oral | 5 | 3 |  |
|  |  |  |  |  |  | Po | Topic | 5 | 26 | 31 |
|  |  |  |  |  | Rheumatism | Po | Topic | 5 | 3 | 3 |
|  |  |  |  |  | Rupture, sprain | Ma | Oral | 5 | 1 |  |
|  |  |  |  |  |  | Po | Topic | 5 | 4 | 5 |
|  |  |  |  |  | Stomach ache | De | Oral | 1 | 1 |  |
|  |  |  |  |  |  | Ma | Oral | 1 | 1 | 2 |
| *Peperomia quadrifolia* (L.) Kunth.  (Piperaceae) | 1538 | Tumin q´een, saqil qeen | He | Ap  7 g | Granos | Ma | Topic | 4 | 1 |  |
|  |  |  |  |  |  |  | Topic | 4 | 1 | 50 |
|  |  |  |  |  | Itch |  | Topic | 4 | 1 | 25 |
|  |  |  |  |  | Skin | Ma | Topic | 4 | 1 | 25 |
| *Persea americana* L.  (Lauraceae) |  | Aguacate, o | Tr | Fr | Abortifacient/menstruation | De | Oral | 9 | 1 | 3 |
|  |  |  |  |  | Gastritis | De | Oral | 1 | 1 | 3 |
|  |  |  |  | Lv  4 g | Alopecia | De | Bath | 4 | 1 | 3 |
|  |  |  |  |  | Headache | Po | Topic | 3 | 1 | 3 |
|  |  |  |  |  | Inflammation | Po | Topic | 5 | 3 | 10 |
|  |  |  |  |  | Mumps | Po | Topic | 12 | 10 | 43 |
|  |  |  |  |  | Muscle Pain after Child Birth | De | Bath | 5 | 1 | 3 |
|  |  |  |  |  | Rupture, sprain | Po | Topic | 5 | 1 | 3 |
|  |  |  |  |  | Stomach ache | De | Oral | 1 | 1 | 3 |
|  |  |  |  | Se  15 g | Birth control | De | Oral | 9 | 3 | 10 |
|  |  |  |  |  | Dysentery | De | Oral | 1 | 2 | 7 |
|  |  |  |  |  | Menstruation | De | Oral | 9 | 2 | 7 |
|  |  |  |  |  | Mumps | Po | Topic | 12 | 3 |  |
| *Petiveria alliaceae* L.  (Phytolacaceae) | 1418 | Apasín | He | Lv  4 g | Chakiq yaj | So | Bath | 10 | 2 |  |
|  |  |  |  |  |  |  | Oral | 10 | 2 | 80 |
|  |  |  |  |  | Spiritual | De | Oral | 10 | 1 | 20 |
| *Phaseolus vulgaris* L.  (Leguminosae) |  | Frijol, quenk | He | Se  5 g | Chakiq yaj | De | Bath | 10 | 2 |  |
|  |  |  |  |  |  |  | Oral | 10 | 1 | 100 |
| *Philodendron tripartitum* (Jacq.) Schott  (Araceae) | 1328 | Manitas | Ep | Lv  10 g | Headache | Po | Topic | 3 | 1 | 50 |
|  |  |  |  |  | Madness | De | Oral | 12 | 1 | 50 |
| *Phyla scaberrima* (Juss. Ex. Pers.) Moldenke  (Lamiaceae) | 1424 | Chojté, Chuejté | Sh | Lv  6 g | Anxiolytic | De | Oral | 12 | 4 |  |
|  |  |  |  |  |  | Inf | Oral | 12 | 1 | 16 |
|  |  |  |  |  | Cough | De | Oral | 2 | 2 | 6 |
|  |  |  |  |  | Depression | De | Oral | 12 | 3 | 10 |
|  |  |  |  |  | Fever | De | Oral | 3 | 12 | 39 |
|  |  |  |  |  | Flu | De | Oral | 2 | 4 |  |
|  |  |  |  |  |  | Po | Topic | 2 | 1 | 16 |
|  |  |  |  |  | Headache | De | Oral | 3 | 3 |  |
|  |  |  |  |  |  | Po | Topic | 3 | 1 | 13 |
| *Phyla x reptans* (Kunth) Greene  (Lamiaceae) |  | Ki´il q´een (hoja dulce) | He | Lv  3 g | Depression | De | Oral | 12 | 1 | 20 |
|  |  |  |  |  | Fever | De | Oral | 3 | 3 | 60 |
|  |  |  |  |  | Headache | De | Oral | 3 | 1 | 20 |
| *Pimenta dioica* (L.) Merr.  (Myrtaceae) | 1501 | Pens | Tr | Fr  2 g | Cough | De | Oral | 2 | 1 | 11 |
|  |  |  |  | Lv  6 g | Fever | De | Bath | 3 | 1 |  |
|  |  |  |  |  |  |  | Oral | 3 | 2 | 33 |
|  |  |  |  |  | Muscle Pain after Child Birth | De | Bath | 5 | 1 |  |
|  |  |  |  |  |  | Inf | Bath | 5 | 1 | 22 |
|  |  |  |  |  | Toothache | De | Oral | 3 | 1 |  |
|  |  |  |  |  |  | Ma | Oral | 3 | 1 | 22 |
|  |  |  |  | Wd | PMS | De | Oral | 9 | 1 | 11 |
| *Pinus* sp.  (Pinaceae) | 1296 | Pino, chaj | Tr | Lv  15 g | Cough | De | Oral | 2 | 3 | 44 |
|  |  |  |  | Re | Rupture, sprain | Po | Topic | 5 | 3 |  |
|  |  | Pino, chaj, Kakichaj (ocote) | Tr | Lv | Rupture, sprain | Po | Topic | 5 | 1 | 28 |
|  |  |  |  | Re | Burns, blister |  | Topic | 4 | 1 | 6 |
|  |  |  |  |  | Cramp | Po | Topic | 5 | 2 | 11 |
|  |  |  |  |  | Inflammation | Po | Topic | 5 | 1 | 6 |
|  |  |  |  |  | Muscle Pain | Po | Topic | 5 | 1 | 6 |
|  |  |  |  |  | Rupture, sprain | Po | Topic | 5 | 1 |  |
|  |  |  |  | Wd | Cough | De | Oral | 2 | 5 |  |
| *Piper auritum* Kunth  (Piperaceae) | 1475 | Santa María, Ob'el, Ub'el | Tr | Lv  10 g | Cramp | Po | Topic | 5 | 1 | 8 |
|  |  |  |  |  | Flu | Po | Topic | 2 | 1 | 8 |
|  |  |  |  |  | Headache | Po | Topic | 3 | 2 | 17 |
|  |  |  |  |  | Inflammation | Po | Topic | 5 | 1 | 8 |
|  |  |  |  |  | Muscle Pain | De | Bath | 5 | 1 |  |
|  |  |  |  |  |  | Po | Topic | 5 | 2 | 25 |
|  |  |  |  |  | Rupture, sprain | Po | Topic | 5 | 1 | 8 |
|  |  |  |  |  | Toothache | De | Oral | 3 | 1 | 25 |
|  |  |  |  | Rt  5 g | Toothache | Ma | Oral | 3 | 2 |  |
| *Piper nigrum* L.  (Piperaceae) |  | Pimienta negra | Sh | Fr  2 g | Dyspepsia | De | Oral | 1 | 1 | 100 |
| *Piper* sp.  (Piperaceae) | 1495 | Moch quej q'en (hembra) | Sh | Lv  10 g | Cramp | Po | Topic | 5 | 1 |  |
|  |  |  |  |  | Muscle Pain | De | Oral | 5 | 1 |  |
|  |  | Moch quej q'en (macho) | Sh | Lv  10 g | Arthritis | De | Oral | 5 | 1 | 20 |
|  |  |  |  |  | Cramp | De | Oral | 5 | 1 | 40 |
|  |  |  |  |  | Muscle Pain | De | Oral | 5 | 1 | 40 |
| *Plantago australis* Lam.  (Plantaginaceae) | 1301 | Llantén, ru´uj raq´ tz´i (lengua de perro) | He | Lv  13 g | Chakiq yaj | De | Bath | 10 | 1 |  |
|  |  |  |  |  |  |  | Oral | 10 | 1 |  |
|  |  |  |  |  |  | Ma | Bath | 10 | 2 |  |
|  |  |  |  |  |  |  | Oral | 10 | 2 | 25 |
|  |  |  |  |  | Cough | De | Oral | 2 | 1 | 4 |
|  |  |  |  |  | Diarrhoea | De | Oral | 1 | 1 | 4 |
|  |  |  |  |  | Gastritis | De | Bath | 1 | 1 |  |
|  |  |  |  |  |  |  | Oral | 1 | 12 |  |
|  |  |  |  |  |  | Inf | Oral | 1 | 1 | 63 |
|  |  |  |  |  | Gout | De | Oral | 12 | 1 | 4 |
|  |  |  |  | Rt | Gastritis | De | Oral | 1 | 1 |  |
| *Plantago major* L.  (Plantaginaceae) | 1391 | Llantén, ru´uj raq´ tz´i (lengua de perro) | He | Lv  13 g | Gastritis | De | Oral | 1 | 2 |  |
|  |  |  |  |  |  | Ma | Oral | 1 | 1 | 100 |
| *Polygala paniculata* L.  (Polygalaceae) | 1347 | Lombriz Q'em | He | Rt  5 g | Vermifuge | De | Oral | 1 | 1 | 100 |
| *Polypodium lindenianum* Kuntze.  (Polypodiaceae) | 1458 | Tisq'en | Ep | Lv  2 g | Diarrhoea | De | Oral | 1 | 3 | 16 |
|  |  |  |  |  | Gastritis | De | Oral | 1 | 1 | 5 |
|  |  |  |  |  | PMS | De | Oral | 9 | 4 | 21 |
|  |  |  |  |  | Stomach ache | De | Oral | 1 | 11 | 58 |
| *Prunus persica* (L.) Stokes  (Rosaceae) | 1502 | Durazno | Tr | Br | Diarrhoea | De | Oral | 1 | 1 | 7 |
|  |  |  |  |  | Toothache | De | Oral | 3 | 1 | 7 |
|  |  |  |  | Lv  5 g | Constipation | De | Oral | 1 | 1 | 3 |
|  |  |  |  |  | Cough | De | Oral | 2 | 1 | 3 |
|  |  |  |  |  | Depression | De | Bath | 12 | 1 |  |
|  |  |  |  |  |  |  | Oral | 12 | 19 |  |
|  |  |  |  |  |  | Inf | Oral | 12 | 1 | 72 |
|  |  |  |  |  | Diarrhoea | De | Oral | 1 | 1 |  |
|  |  |  |  |  | Flu | De | Oral | 2 | 1 | 3 |
|  |  |  |  |  | Muscle Pain after Child Birth | Inf | Bath | 5 | 1 | 3 |
|  |  |  |  | Rt | Toothache | De | Oral | 3 | 1 |  |
| *Psidium guajava* L.  (Myrtaceae) | 1428 | Guayaba, patá | Tr | Br  15 g | Abortifacient/menstruation | De | Oral | 9 | 1 | 2 |
|  |  |  |  |  | Diarrhoea | De | Oral | 1 | 5 | 31 |
|  |  |  |  |  | Dysentery | De | Oral | 1 | 5 | 14 |
|  |  |  |  |  | Dyspepsia | De | Oral | 1 | 1 | 6 |
|  |  |  |  |  | Stomach ache | De | Oral | 1 | 1 | 10 |
|  |  |  |  |  | Toothache | De | Oral | 3 | 1 | 2 |
|  |  |  |  | Lv  7 g | Cough | De | Oral | 2 | 1 | 2 |
|  |  |  |  |  | Depression | De | Oral | 12 | 6 |  |
|  |  |  |  |  |  | Inf | Oral | 12 | 1 | 14 |
|  |  |  |  |  | Diarrhoea | De | Oral | 1 | 11 |  |
|  |  |  |  |  | Dysentery | De | Oral | 1 | 2 |  |
|  |  |  |  |  | Dyspepsia | De | Oral | 1 | 2 |  |
|  |  |  |  |  | Fever | De | Oral | 3 | 1 | 2 |
|  |  |  |  |  | Gastritis | De | Oral | 1 | 1 | 2 |
|  |  |  |  |  | Granos | Po | Topic | 4 | 1 | 2 |
|  |  |  |  |  | Itch | De | Bath | 4 | 4 | 8 |
|  |  |  |  |  | Muscle Pain after Child Birth | De | Bath | 5 | 1 |  |
|  |  |  |  |  |  | Inf | Bath | 5 | 1 | 4 |
|  |  |  |  |  | Stomach ache | De | Oral | 1 | 4 |  |
|  |  |  |  |  | Weakness | Inf | Bath | 5 | 1 | 2 |
| *Quercus* sp.  (Fagaceae) | 1559 | Encino, Jí | Tr | Br  60 g | Diarrhoea | De | Oral | 1 | 2 | 15 |
|  |  |  |  |  | Dysentery | De | Oral | 1 | 1 | 8 |
|  |  |  |  |  | Gastritis | De | Oral | 1 | 2 | 15 |
|  |  |  |  |  | Headache | De | Bath | 3 | 1 | 8 |
|  |  |  |  |  | Menstruation | De | Oral | 9 | 1 | 8 |
|  |  |  |  |  | Mu | De | Bath | 10 | 1 | 8 |
|  |  |  |  |  | Muscle Pain | De | Bath | 5 | 1 | 8 |
|  |  |  |  |  | Stomach ache | De | Oral | 1 | 1 | 8 |
|  |  |  |  |  | Toothache | De | Oral | 3 | 2 | 15 |
|  |  |  |  | Lv | Muscle Pain after Child Birth | Inf | Bath | 5 | 1 | 8 |
| *Raphanus sativus* L.  (Brassicaceae) |  | Rábano | He | Lv  7 g | Menstruation | De | Oral | 9 | 1 | 100 |
| *Renealmia aromatica* (Aubl.) Griseb  (Zingiberaceae) | 1455 | Tib´ej Tz´i (comida de perro) | He | Lv  21 g | Arthritis | Po | Topic | 5 | 1 | 8 |
|  |  |  |  |  | Chakiq yaj | Ma | Bath | 10 | 1 |  |
|  |  |  |  |  |  |  | Oral | 10 | 1 | 15 |
|  |  |  |  |  | Inflammation | Po | Topic | 5 | 2 | 15 |
|  |  |  |  |  | Muscle Pain | Po | Topic | 5 | 3 | 23 |
|  |  |  |  |  | Rupture, sprain | Po | Topic | 5 | 2 | 15 |
|  |  |  | Sh | Lv | Headache | Po | Topic | 3 | 2 | 15 |
|  |  |  |  |  | Muscle Pain after Child Birth | Po | Topic | 5 | 1 | 8 |
| *Rosa* sp. |  | Rosas | Sh | Fl  6 g | Granos | Ma | Topic | 4 | 1 | 100 |
| *Rosmarinus officinalis* L.  (Lamiaceae) |  | Romero, Xac chaj q'en | Sh | Lv  5-8 g | Antiemetic | De | Oral | 1 | 1 | 3 |
|  |  |  |  |  | Depression | De | Oral | 12 | 1 | 3 |
|  |  |  |  |  | Diarrhoea | De | Oral | 1 | 1 | 3 |
|  |  |  |  |  | Mu | Bu | Inhalated | 10 | 18 | 58 |
|  |  |  |  |  | Rotavirus | Bu | Inhalated | 12 | 1 | 3 |
|  |  |  |  |  | Scare | Bu | Inhalated | 10 | 2 |  |
|  |  |  |  |  |  | De | Oral | 10 | 1 | 10 |
|  |  |  |  |  | Stomach ache | De | Oral | 1 | 3 | 10 |
|  |  |  |  |  | Weakness | Bu | Inhalated | 5 | 1 |  |
|  |  |  |  |  |  | De | Bath | 5 | 2 | 10 |
| *Rumex obtusifolius* L.  (Polygonaceae) | 1407 | Lengua de vaca | He | Lv  15 g | Varicose vains | Ma | Topic | 6 | 1 | 100 |
| *Ruta graveolens* L.  (Rutaceae) |  | Ruda | He | Lv  20 g | Blood pressure | Inf | Oral | 6 | 1 | 2 |
|  |  |  |  |  | Chakiq yaj | De | Bath | 10 | 16 |  |
|  |  |  |  |  |  |  | Oral | 10 | 2 |  |
|  |  |  |  |  |  | Inf | Bath | 10 | 3 |  |
|  |  |  |  |  |  | Ma | Bath | 10 | 1 |  |
|  |  |  |  |  |  |  | Oral | 10 | 1 |  |
|  |  |  |  |  |  | So | Bath | 10 | 4 |  |
|  |  |  |  |  |  |  | Oral | 10 | 2 | 56 |
|  |  |  |  |  | Depression | De | Bath | 12 | 1 |  |
|  |  |  |  |  |  |  | Oral | 12 | 11 |  |
|  |  |  |  |  |  | Inf | Oral | 12 | 1 | 25 |
|  |  |  |  |  | Fever |  | Topic | 3 | 1 | 2 |
|  |  |  |  |  | Headache | Po | Topic | 3 | 1 | 2 |
|  |  |  |  |  | Mal de ojo | De | Bath | 10 | 3 |  |
|  |  |  |  |  |  |  | Oral | 10 | 1 |  |
|  |  |  |  |  |  |  | Topic | 10 | 2 | 12 |
|  |  |  |  |  | Stomach ache | De | Oral | 1 | 1 | 2 |
| *Salmea scandens* (L.) DC.  (Compositae) | 1295 | Ra´il K´aam, Wo k´aam, Ik k'aam | Sh | Lv  10-25 g | Acne | Ma | Topic | 4 | 1 | 2 |
|  |  |  |  |  | Blood cleaner | De | Oral | 6 | 2 |  |
|  |  |  |  |  |  | Inf | Oral | 6 | 1 | 7 |
|  |  |  |  |  | Burns, blister | Po | Topic | 4 | 1 | 2 |
|  |  |  |  |  | Diarrhoea | De | Oral | 1 | 1 | 2 |
|  |  |  |  |  | Dysentery | De | Oral | 1 | 1 | 2 |
|  |  |  |  |  | Gastritis | De | Oral | 1 | 3 |  |
|  |  |  |  |  |  |  | Oral | 1 | 1 | 9 |
|  |  |  |  |  | Granos | De | Bath | 4 | 3 |  |
|  |  |  |  |  |  | Ma | Topic | 4 | 1 |  |
|  |  |  |  |  |  | Po | Topic | 4 | 3 |  |
|  |  |  |  |  |  |  | Topic | 4 | 1 | 19 |
|  |  |  |  |  | Headache | De | Oral | 3 | 1 | 2 |
|  |  |  |  |  | Inflammation | De | Bath | 5 | 3 | 7 |
|  |  |  |  |  | Itch | De | Bath | 4 | 6 |  |
|  |  |  |  |  |  | Po | Topic | 4 | 1 |  |
|  |  |  |  |  |  |  | Topic | 4 | 2 | 21 |
|  |  |  |  |  | Mal de ojo | De | Bath | 10 | 1 | 2 |
|  |  |  |  |  | Measles | De | Bath | 4 | 1 | 2 |
|  |  |  |  |  | Mu | De | Bath | 10 | 1 | 2 |
|  |  |  |  |  | Muscle Pain after Child Birth | Inf | Bath | 5 | 1 | 2 |
|  |  |  |  |  | Rupture, sprain | Po | Topic | 5 | 1 | 2 |
|  |  |  |  |  | Skin | De | Bath | 4 | 1 | 2 |
|  |  |  |  |  | Stomach ache | De | Oral | 1 | 1 |  |
|  |  |  |  |  |  |  | Topic | 1 | 1 | 5 |
|  |  |  |  |  | Toothache | De | Oral | 3 | 1 | 2 |
|  |  |  |  |  | Urinary infection | De | Oral | 7 | 1 | 2 |
|  |  |  |  | Rt | Muscle Pain | De | Oral | 5 | 1 | 2 |
| *Salvia urica* Epling  (Lamiaceae) | 1285 | Chu'Onon, Ju Onon | He | Lv  4 g | Toothache | Inf | Oral | 3 | 1 | 100 |
| *Sansevieria trifasciata* Prain  (Asparagaceae) | 1424 | Curarina | He | Lv  15 g | Antiophidic | De | Oral | 12 | 2 | 50 |
|  |  |  |  |  | Dermatitis | De | Oral | 4 | 1 | 25 |
|  |  |  |  |  | Antirabies | Ma | Oral | 12 | 1 | 25 |
| *Sechium edule* (Jacq.) Sw.  (Cucurbitaceae) | 1334 | Guisquil, Chima´ | Vi | Fr | Burns, blister | Ma | Topic | 4 | 1 |  |
|  |  |  |  |  |  | Po | Topic | 4 | 4 |  |
|  |  |  |  |  |  |  | Topic | 4 | 4 | 50 |
|  |  |  |  | Lv  40 g | Chakiq yaj | De | Bath | 10 | 1 | 6 |
|  |  |  |  |  | Heal |  | Topic | 4 | 1 | 6 |
|  |  |  |  |  | heat/sweat in child | De | Bath | 10 | 3 | 17 |
|  |  |  |  |  | Mal de ojo | De | Bath | 10 | 1 |  |
|  |  |  |  |  |  |  | Topic | 10 | 3 | 22 |
| *Senecio* sp.  (Compositae) | 1513 | Hoja de queso | Sh | Lv  15 g | Itch | De | Bath | 4 | 1 | 100 |
| *Sida rhombifolia* L.  (Malvaceae) | 1291 | Escobillo, Saquil Mesbe' | Sh | Ap  50 g | Alopecia | De | Bath | 4 | 1 |  |
|  |  |  |  |  |  | Ma | Bath | 4 | 3 |  |
|  |  |  |  |  |  |  | Topic | 4 | 1 |  |
|  |  |  |  |  |  | So | Bath | 4 | 1 | 75 |
|  |  |  |  | Lv  30 g | asthma | De | Bath | 2 | 1 |  |
|  |  |  |  |  |  |  | Inhalation | 2 | 1 |  |
|  |  |  |  |  |  |  | Oral | 2 | 1 | 19 |
|  |  |  |  |  | Facilitate birth | De | Oral | 9 | 1 | 6 |
|  |  |  |  | Rt | Alopecia | Ma | Bath | 4 | 6 |  |
| *Siparuna thecaphora* (Poepp. & Endl.) A. DC.  (Siparunaceae) | 1346 | Chu Ché | Tr | Lv  6 g | asthma | De | Oral | 2 | 1 | 4 |
|  |  |  |  |  | Cough | De | Oral | 2 | 6 |  |
|  |  |  |  |  |  | Inf | Bath | 2 | 1 | 26 |
|  |  |  |  |  | Depression | De | Bath | 12 | 1 |  |
|  |  |  |  |  |  |  | Oral | 12 | 1 | 7 |
|  |  |  |  |  | Flu | De | Bath | 2 | 1 |  |
|  |  |  |  |  |  |  | Oral | 2 | 2 |  |
|  |  |  |  |  |  | Inf | Inhalation | 2 | 1 | 15 |
|  |  |  |  |  | Headache | Po | Topic | 3 | 8 | 30 |
|  |  |  |  |  | Inflammation | Po | Topic | 5 | 1 | 4 |
|  |  |  |  |  | Muscle Pain | Po | Topic | 5 | 1 | 4 |
|  |  |  |  |  | Muscle Pain after Child Birth | Inf | Bath | 5 | 1 | 4 |
|  |  |  |  |  | Nasal congestion | Inf | Inhalation | 2 | 1 | 4 |
|  |  |  |  |  | Rupture, sprain | Po | Topic | 5 | 1 | 4 |
| *Smallanthus maculatus* var. *maculatus* (Cav.) H. Rob.  (Compositae) | 1427 | Ax, Arnica | He | Lv  4 g | Chakiq yaj | Inf | Bath | 10 | 1 | 1 |
|  |  |  |  |  | Gastritis | De | Oral | 1 | 1 | 3 |
|  |  |  |  |  | Gout | De | Oral | 12 | 1 |  |
|  |  |  |  |  |  | Po | Topic | 12 | 1 | 3 |
|  |  |  |  |  | Itch | De | Bath | 4 | 1 |  |
|  |  |  |  |  |  | Ma | Topic | 4 | 1 | 3 |
|  |  |  |  |  | Wounds | Po | Topic | 4 | 1 | 90 |
|  |  |  |  | Lv, Juice | Wounds | Po | Topic | 4 | 2 |  |
|  |  |  |  | Lv, St Juice | Wounds | Po | Topic | 4 | 62 |  |
|  |  |  |  | Rt | Gastritis | De | Oral | 1 | 1 |  |
|  |  |  |  | St | Wounds | De | Topic | 4 | 1 |  |
| *Solanum americanum* Miller.  (Solanaceae) | 1446 | Macuy | He | Fr  4 g | Menstruation | Inf | Oral | 9 | 1 | 30 |
|  |  |  |  | Lv  40 g | Anemia | De | Oral | 12 | 1 | 10 |
|  |  |  |  |  | Granos | De | Bath | 4 | 1 | 10 |
|  |  |  |  |  | heat/sweat in child | De | Bath | 10 | 1 |  |
|  |  |  |  |  |  | Po | Topic | 10 | 1 | 20 |
|  |  |  |  |  | Itch | Ma | Topic | 4 | 1 | 10 |
|  |  |  |  |  | Menstruation | Inf | Oral | 9 | 2 |  |
|  |  |  |  |  | Vaginal infection | De | Bath | 9 | 1 |  |
|  |  |  |  |  |  |  | Wash | 9 | 1 | 20 |
| *Solanum aphyodendron* S. Knapp  (Solanaceae) | 1284 | Sak Yol | Sh | Lv  20 g | Chickenpox | Ma | Topic | 4 | 1 | 25 |
|  |  |  |  |  | Dermatobia hominis | Ma | Topic | 4 | 1 | 25 |
|  |  |  |  |  | Measles | Ma | Topic | 4 | 1 | 25 |
|  |  |  |  |  | Mumps | Ma | Topic | 12 | 1 | 25 |
| *Solanum rudepannum* Dunal  (Solanaceae) | 1283 | Paj Kix, Pajch, tun qix | Sh | Lv  40 g | Chakiq yaj | De | Bath | 10 | 1 | 100 |
| *Solanum* sp.  (Solanaceae) |  | Macuy | He | Lv  15 g | Fever | Po | Topic | 3 | 1 | 100 |
| *Sonchus oleraceus* (L.) L  (Compositae) | 1294 | Lechuguilla | He | Lv  5 g | Diabetes | De | Oral | 8 | 1 | 100 |
| *Spananthe paniculata* Jacq.  (Apiaceae) | 1563 | Valeriana | He | Rt | Anxiolytic | De | Oral | 12 | 1 | 100 |
| *Spermacoce laevis* Lam.  (Rubiaceae) | 1436 | Rubiaceae flores blancas | He | Ap  4 g | Menstruation | De | Oral | 9 | 1 | 33 |
|  |  |  |  |  | Urinary infection | De | Oral | 7 | 1 | 33 |
|  |  |  |  | Lv | Vaginal infection | De | Oral | 9 | 1 | 33 |
| *Spondias* sp.  (Anacardiaceae) | 1302 | Jocote, rum | Tr | Lv  5 g | Depression | De | Oral | 12 | 2 | 100 |
| *Stachytarpheta cayennensis* (Rich.) Vahl.  (Verbenaceae) | 1396 | Verbena, Xk´ot aaqam (heces de cotuza cotuza) | He | Lv  2 g | Conjunctivitis |  | Topic | 12 | 1 | 7 |
|  |  |  |  |  | Diarrhoea | De | Oral | 1 | 4 | 29 |
|  |  |  |  |  | Stomach ache | De | Oral | 1 | 9 | 64 |
| *Syzygium aromaticum* (L.) Merr. & L.M.Perry  (Myrtaceae) |  | Clavo | Tr | Fl  2 g | Cough | De | Oral | 2 | 2 | 40 |
|  |  |  |  |  | Dyspepsia | De | Oral | 1 | 1 | 40 |
|  |  |  |  |  | PMS | De | Oral | 9 | 1 | 20 |
|  |  |  |  | Lv | Dyspepsia | De | Oral | 1 | 1 |  |
| *Tagetes erecta* L.  (Compositae) | 1388 | Tuus | He | Fl  58 g | Chakiq yaj | De | Bath | 10 | 1 | 50 |
|  |  |  |  |  | Gastritis | De | Oral | 1 | 1 | 17 |
|  |  |  |  | Lv  58 g | Chakiq yaj | Ma | Bath | 10 | 1 |  |
|  |  |  |  |  |  |  | Oral | 10 | 1 |  |
|  |  |  |  |  | heat/sweat in child | De | Bath | 10 | 1 |  |
|  |  |  |  |  |  | Po | Topic | 10 | 1 | 33 |
| *Tagetes lucida* Cav.  (Compositae) |  | Pericón | He | Ap  3 g | Diarrhoea | De | Oral | 1 | 1 | 11 |
|  |  |  |  |  | Dyspepsia | De | Oral | 1 | 1 | 11 |
|  |  |  |  |  | Menstruation | Inf | Oral | 9 | 1 | 11 |
|  |  |  |  |  | PMS | De | Oral | 9 | 3 | 33 |
|  |  |  |  |  | Stomach ache | De | Oral | 1 | 3 | 33 |
| *Teucrium vesicarium* Mill.  (Lamiaceae) | 1452 | Kaawakax, Xkaawakax | He | Lv  4 g | Diarrhoea | De | Oral | 1 | 4 | 36 |
|  |  |  |  |  | Dyspepsia | De | Oral | 1 | 2 | 18 |
|  |  |  |  |  | Gastritis | De | Oral | 1 | 1 | 9 |
|  |  |  |  |  | PMS | De | Oral | 9 | 1 | 9 |
|  |  |  |  |  | Stomach ache | De | Oral | 1 | 3 | 27 |
| *Theobroma cacao* L.  (Malvaceae) |  | Cacao, Kakaw | Tr | Se  8 g | Alopecia | Ma | Bath | 4 | 2 | 13 |
|  |  |  |  |  | Chakiq yaj | De | Bath | 10 | 5 |  |
|  |  |  |  |  |  |  | Oral | 10 | 2 |  |
|  |  |  |  |  |  | Inf | Bath | 10 | 1 |  |
|  |  |  |  |  |  | Ma | Bath | 10 | 1 |  |
|  |  |  |  |  |  |  | Oral | 10 | 1 | 67 |
|  |  |  |  |  | Fever | Ma | Bath | 3 | 1 |  |
|  |  |  |  |  |  |  | Oral | 3 | 1 | 13 |
|  |  |  |  |  | Scare | De | Bath | 10 | 1 | 7 |
| *Thymus* sp.  (Lamiaceae) |  | Tomillo | Sh | Lv  1 g | Cough | De | Oral | 2 | 2 | 100 |
| *Tradescantia zebrina* var. *zebrina* Bosse  (Commelinaceae) | 1400 | Hierba de pollo | He | Lv  15 g | Menstruation | De | Oral | 9 | 1 | 100 |
| *Triumfetta bogotensis* DC.  (Malvaceae) | 1486 | Ruk Max, piojo de mico | Sh | Lv  6 g | Childbirth | De | Oral | 9 | 2 | 50 |
|  |  |  |  |  | Diarrhoea | De | Oral | 1 | 2 | 25 |
|  |  |  |  |  | Dysentery | De | Oral | 1 | 1 | 13 |
|  |  |  |  |  | Stomach ache | De | Oral | 1 | 1 | 13 |
|  |  |  |  | Rt  5 g | Childbirth | De | Oral | 9 | 1 |  |
|  |  |  |  | St | Childbirth | De | Oral | 9 | 1 |  |
| *Valeriana scandens* var. *candonella* (Gardner) C.A. Mull.  (Caprifoliaceae) | 1539 | Valeriana | He | Ap  6 g | Tenia pedis /Smelly feet | Ma | Topic | 4 | 1 | 100 |
| *Verbena litoralis* Kunth.  (Verbenaceae) | 1308 | Verbena, K'ot A' Q'am | He | Lv  3 g | Cough | De | Oral | 2 | 3 | 75 |
|  |  |  |  |  | Fever | De | Oral | 3 | 1 | 25 |
| *Vernonanthura patens* (Kunth) H. Rob  (Compositae) | 1305 | Semem | Tr | Lv  2 g | Depression | De | Oral | 12 | 2 | 100 |
| *Vismia camparaguey* Sprague & L. Riley  (Hypericaceae) | 1333 | Camparaguay, Camparcual | Tr | Br  20 g | Toothache | De | Oral | 3 | 1 |  |
|  |  |  |  |  |  | Ma | Oral | 3 | 2 | 60 |
|  |  |  |  | Lv  4 g | Hepatitis | De | Oral | 12 | 2 | 40 |
| *Yucca elephantipes* Regel.  (Asparagaceae) | 1349 | Izote, Kuq'il | Tr | St  15 g | Depression | De | Oral | 12 | 1 | 100 |
| *Zea mays* L.  (Poaceae) |  | Ixim | He | Fl  2 g | Blood cleaner | De | Oral | 6 | 1 | 8 |
|  |  |  |  |  | Hepatitis | De | Oral | 12 | 1 | 8 |
|  |  |  |  |  | Urinary infection | De | Oral | 7 | 9 | 75 |
|  |  |  |  | Fr | Dyspepsia | Bu | Oral | 1 | 1 | 8 |
| *Zingiber officinale* Roscoe  (Zingiberaceae) |  | Gengibre | He | St  4 g | Dyspepsia | De | Oral | 1 | 1 | 100 |

The plants without number of voucher could not be found in the gardens, roadsides or on remote sites, rather they were buyed in the markets.  Abbreviations: Form: Sh, Shrub; He, herb;  Tr, Tree; Ep, Epiphyt;  Vi, I came. Used part: Lv, leaves;  seed;  Rt, root;   Br, bark;   St, stem;   Cat, Cataphylls; Fr; Fl, fruits, flowers;  AP, Aerial parts,  Re, resine; Wd, wood.  Wanted: PMS, premenstrual syndrome.  Preparation: Po, poultice; Inf, infusion; Ma, macerated; De, decoction; N.A., not apply; So, soak; Bu, burned; Fr, fried; Ch, chew; Alc, alcohol; Pre, pressed; Ju, juice. Cat: categories for the Fic. Mu: number of mentions. Fi: fidelity level of Friedman.
