# Supplementary material for: Ethnopharmacological Field Study of Three Q'eqchi Communities in Guatemala
Source: Front Pharmacol. 2018 Nov 6;9:1246. doi: 10.3389/fphar.2018.01246 (PMC6240767; doi:10.3389/fphar.2018.01246)
Supplement: Supplementary file 2 [file Image_1.pdf]

Location Map for Communities

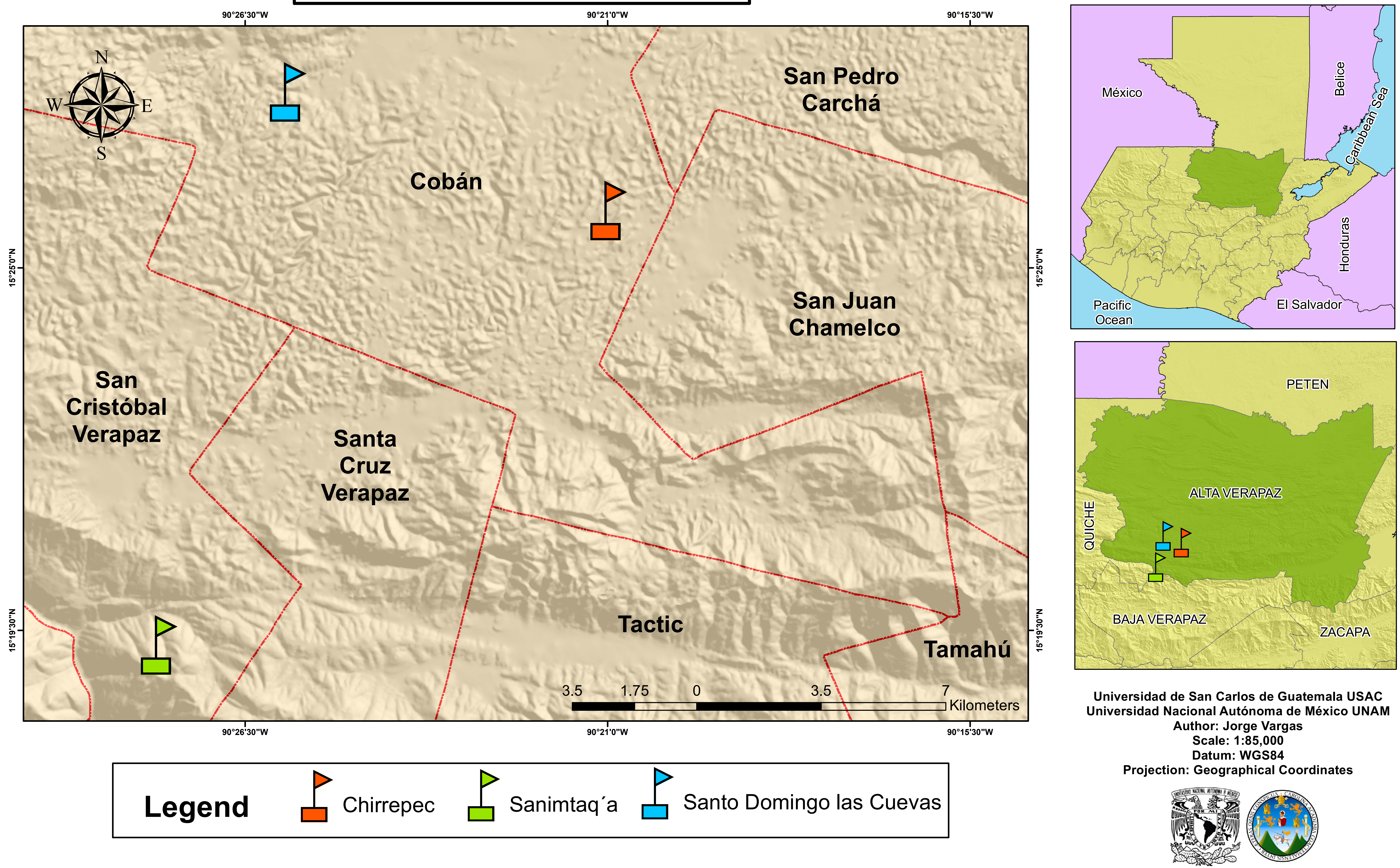

Universidad de San Carlos de Guatemala USAC
Universidad Nacional Autónoma de México UNAM
Author: Jorge Vargas
Scale: 1:85,000
Datum: WGS84
Projection: Geographical Coordinates
